# Combining Proteomics and Metabolomics to Analyze the Effects of Spaceflight on Rice Progeny

**DOI:** 10.3389/fpls.2022.900143

**Published:** 2022-06-21

**Authors:** Deyong Zeng, Jie Cui, Yishu Yin, Cuihong Dai, Haitian Zhao, Chen Song, Shuanghong Guan, Dayou Cheng, Yeqing Sun, Weihong Lu

**Affiliations:** ^1^Department of Food Science and Engineering, School of Chemistry and Chemical Engineering, Harbin Institute of Technology, Harbin, China; ^2^National and Local Joint Engineering Laboratory for Synthesis, Transformation and Separation of Extreme Environmental Nutrients, Harbin Institute of Technology, Harbin, China; ^3^The Intelligent Equipment Research Center for the Exploitation of Characteristic Food & Medicine Resources, Chongqing Research Institute, Harbin Institute of Technology, Chongqing, China; ^4^Institute of Environmental Systems Biology, Dalian Maritime University, Dalian, China

**Keywords:** rice, iTRAQ, metabolomics, space flight, SJ-10 returning satellite

## Abstract

Spaceflight is a special abiotic stress, the biological effect mechanism of which on contemporary rice has been clarified, However, its effect on offspring rice was still unclear. In order to understand the response mechanism of F2 generation plants to space flight, this study used SJ-10 recoverable satellite to carry DN423 rice seeds for 12.5 days in orbit flight. After returning to the ground, the plants were then planted to F2 generation to explore the biological effect mechanism. Our research showed that in the F2 generation of TLS, the rice plant height of the space flight group increased by 33.8%, the ear length and thousand-grain weight decreased by 9.7 and 4.6%, respectively, and the grain number per panicle increased by 6.5%. Moreover, related proteins that control changes in agronomic traits have been identified. The changes of MDA, H_2_O_2_, soluble sugar, electron leakage and antioxidant enzyme activity confirmed the stress response in F2 generation plants. ITRAQ and LC-MS technology were used to reveal the change pattern of protein levels and metabolite levels in F2 generation plants, 389 and 405 proteins were identified as differentially abundant proteins in TLS and TS, respectively. In addition, there were 124 and 125 metabolites that changed during these two periods. The proteome and metabolome result further confirmed that the F2 generation plants still retained the memory of space flight stress, and retained the memory of space flight stress through genome instability. Oxidative stress signals activated sugar signals to rebuild metabolic networks to adapt to space flight stress. The reconstruction of energy metabolism, amino acid metabolism, phenylalanine metabolism, and flavonoid metabolism played an important role in the process of adapting to space flight stress. The results of this study broaden the perspective of space biological effects and provide a basis for studying the effects of abiotic stress on plant progeny.

## HIGHLIGHTS

- Spaceflight changed the agronomic traits of F2 generation rice.
- The F2 generation rice retained the memory of stress to space flight, which was activated by ROS.
- The assembly process of mitochondrial complex III was blocked, which was the main reason for the increase of ROS in F2 generation rice.
- The response of F2 rice to spaceflight stress caused changes in the processes of sugar metabolism, amino acid metabolism, and energy metabolism.

## Introduction

Space environment has the characteristics of strong radiation, high vacuum, microgravity, changing magnetic field and so on. Different from the earth environment, space environment has a strong mutagenic effect on organisms. In the past few decades, scientists have tried to use various returnable satellites, the International Space Station, spacecrafts and other aircrafts to carry plants for research in space life sciences. From the change of plant agronomic traits to the research of molecular level, the influence of spaceflight on plants has been revealed gradually. In recent years, omics techniques have been used to explore the mechanism of plant mutations caused by spaceflight. However, these studies have rarely reported the simultaneous use of two or more omics to analyze the effects of spaceflight on plants.

It has been proved by Vaulina et al. (1984) that spaceflight can cause chromosome aberration, gene deletion and recombination of crops, thus causing genetic material variation. Moreover, crops adapted to the effects of spaceflight by adjusting their own metabolic network (Paul et al., 2017), which was induced by oxidative stress and heat stress (Manian et al., 2021). The current research conclusions showed that spaceflight has caused changes in plant lipid peroxidation, antioxidant enzymes, energy metabolism, signal transduction, protein synthesis, cell wall biosynthesis and other processes (Matía et al., 2010; Ferl et al., 2015; Choi et al., 2019). At the same time, studies have found that spaceflight increased the content of soluble sugar, glucose, fructose, sucrose and total starch (Mortley et al., 2008). Our previous study confirmed that spaceflight stress affected different growth periods in contemporary rice. After spaceflight, contemporary plants have undergone significant changes in metabolic pathways such as energy metabolism, amino acid metabolism, sugar metabolism, and vitamin B6 metabolism. It is believed that these changes were caused by the disruption of ROS balance in rice after spaceflight (Deyong et al., 2020; Zeng et al., 2020, 2021). Recent studies have shown that abiotic stress has genetic effects across generations (Kumar et al., 2015). As a special abiotic stress, spaceflight also has intergenerational genetic effects, and how the offspring rice adapts to the intergenerational genetic effects by adjusting its own metabolic network needs to be reported. Over the past 20 years, our team has carried 50 different varieties of rice seeds by using China’s recoverable satellites and spacecraft. After returning to the ground for planting, we analyzed the biological effects and confirmed that short-term low orbit flight can also cause the biological effects on rice seeds (Sun et al., 2019). Our team planted rice seeds to M2 generation after spaceflight, and found mutation sites in the DNA of M2 generation plants, whose rate was between 0.05 and 0.52% (Yu et al., 2007). The plant height, heading date, leaf color, leaf shape, flag leaf angle, awn, panicle length, panicle shape, maturity and other agronomic traits of plants in the M2 generation also changed significantly (Wei et al., 2006; Yu et al., 2007). Our team’s early results suggested that the impact of spaceflight on rice seeds were heritable, but how can M2 plants respond to the impact of spaceflight by regulating their own metabolic network? Our team used two-dimensional polyacrylamide gel electrophoresis (2D-PAGE) to analyze the proteome of the M2 generation plants. Proteins involved in amino acid and derivative metabolism, pentose phosphate shunting, stress response, seed maturation, protein folding, glycolysis, lipid biosynthesis, glycogen biosynthesis and TCA cycle were differentially expressed in M2 generation plants (Sun et al., 2019). However, the ability of 2D-PAGE to identify differentially abundant proteins was limited, so it may greatly limit our understanding of how M2 generation plants respond to spaceflight. At the same time, there was no report on the changes in the metabolic map of the M2 generation plants after spaceflight.

Clarifying the response mode of progeny plants to spaceflight is helpful to reveal the mechanism of spaceflight on crop genetic effects, which is crucial to the study of the mechanism of biological effects caused by spaceflight. In this study, DN423 seeds were placed in biological irradiation box A (BRB-A) for space flight. The surface material of the BRB is aluminum with an average thickness of 2.5 mm. The space radiation measurement module and the model organism module are included in BRB-A. Rice seeds were immobilized in the model organism module of BRB-A, and there was no mechanical collision between rice seeds and BRB-A. BRB-A was fixed on the -Y axis of the SJ-10 satellite for space flight (Sun et al., 2019). The SJ-10 returnable satellite was launched at 01:38 on April 6, 2016, with an orbital altitude of 252 kilometers, an inclination of 42°, and an orbital flight of 12.5 days (Sun et al., 2019). The g-profile during launch was as follows: the first-level maximum static overload was 4.8 g in flight for 150 s; the second-level maximum static overload was 6.0 g in flight for 180 s (Xu et al., 2018). After entering orbit, the radiation dose rate measured by TLD700 was 0.075 ± 0.005 mGy/d, and the total LET radiation dose measured by CR-39 was 0.970 ± 0.055 mGy (Zhou et al., 2018), the load temperature was 20.6–23.6°C, and the gravity level was 10^–4^∼10^–6^ g. During landing, the temperature of the recovery cabin was controlled at 22 ± 2°C, and the landing speed was 12.5 m/s (Zhao et al., 2019). First, the agronomic traits of the F2 generation plants were analyzed, and then the changes in the redox state in the body were discussed. iTRAQ proteomics and non-targeted metabolomics were used to explain the response mode of F2 generation plants to spaceflight, and finally using RT-qPCR to verify the results of the proteomics and metabolomics. This study broadens our understanding of spatial biological effects, and also provides a basis for subsequent research on how F2 generation plants retain memories of abiotic stress. At TLS and TS, we assessed the plant height of rice. At TS, we assessed tiller number. At maturity, we assessed the rice’s, setting percentage, grain number per panicle, thousand seed weight. In addition, we also evaluated physiological indicators such as H_2_O_2_, MDA, soluble sugar contents, electrolyte leakage rate, APX activity, SOD activity, CAT activity, and POD activity of rice during TLS and TS, respectively.

## Materials and Methods

### Plant Materials

The cultivation of rice material was used as described by Yu et al. (2007) and Deyong et al. (2020). Briefly, the DN423 rice seeds were carried by the SJ-10 returnable satellite for 12.5 days of spaceflight, and then planted after 400 rice seeds returning to the ground. When sampling at the tillering stage of the F1 generation, we randomly selected 30 rice plants for sampling and labeled them. After the F1 generation matures, the 30 rice seeds were harvested, and 400 F1 generation seeds were randomly selected for planting to obtain the F2 generation. The control group was sampled and planted in the same way. The F2 generation rice was planted in Wuchang City, Heilongjiang Province in April 2017. For the F2 generation plants, we harvested the leaves at the three-leaf stage (TLS) and the tillering stage (TS), and stored them at −80°C for further analysis. In F2 generation plants, five plants were randomly selected and their leaves were mixed to obtain a single biological replicate. In the metabolomics experiment, we performed six biological replicates, while we performed three biological replicates in others. Use SP2 to represent the F2 generation plant of the rice seed after spaceflight, and CK to represent the F2 generation plant of the rice seed without spaceflight. Seeds of DN423 (*Oryza sativa L*.) were provided and certified by the Agricultural College of Northeast Agricultural University.

### Detection of H_2_O_2_ and MDA Formation in Leaves

The H_2_O_2_ was measured according to the method described by Velikova et al. (2000). Simply, take about 200 mg of rice leaves were taken and homogenized in 2 mL of 0.1% (w/v) trichloroacetic acid. The homogenate was centrifuged at 4°C (12,000 × g for 15 min). Subsequently, take 0.25 mL of the homogenate and add 0.75 mL buffer solution to it, consisting of 10 mM potassium phosphate (pH 7.0) and 1 mL 1 M potassium iodide (KI), with a final volume of 2.0 mL in each tube. The absorbance value was measured at 390 nm and calculate the H_2_O_2_ content.

The determination of Lipid peroxidation was carried out according to the method described by Heath and Packer (1968). About 200 mg of rice leaves were homogenized in 2 mL of 0.1% (w/v) trichloroacetic acid, and then centrifuged at 4°C (12,000 × g for 15 min). Take 0.5 mL of the supernatant and add it to 1.5 mL of thiobarbituric acid, and then incubated at 90°C for 20 min. The reaction solution was put on ice to terminate the reaction, and the MDA content was calculated by reading the absorbance at 535 nm and 600 nm.

### Detection of Electrolyte Leakage Rate

The electrolyte leakage rate (EL) was measured according to the method described by Lutts et al. (1996). Simply, 0.5 g of rice leaves were placed in 25 mL of deionized water, at 25°C for 3 h, and the measured conductivity was recorded as H1. Then boiled for 10 min, cooled to 25°C and measured the conductivity, which was recorded as H2, and calculated EL according to the following formula:


EL=H1/H2×100%

### Detection of Soluble Sugar Content

The soluble sugar content was determined according to the method described in Bailey (1958). Simply, 100 mg of rice leaves were weighed, 5 mL of 80% ethanol was added, and extracted at 80°C for 30 min. Then the sample was centrifuged to collect the supernatant. The extraction was repeated three times. Next, anthrone reagent was added to the supernatant and incubated at 95°C for 20 min for color reaction. After completion, the reaction was stopped on ice and the absorbance was measured at 620 nm.

### Determination of Antioxidant Enzyme Activity

About 0.5 g rice leaves were placed in a homogenization tube, 5 mL phosphate buffer (100 mM, pH 7.4) was added, ground thoroughly on ice, and then centrifuged at 11,000 *g* for 15 min at 4°C to collect. The supernatant was used as a crude enzyme extract for subsequent enzyme activity analysis. The peroxidase (POD) activity was measured according to the method described by Castillo et al. (1984). The catalase (CAT) activity was measured according to the method described in Aebi (1984). The activity of SOD and APX was measured according to the method described by Foreman et al. (2003).

### Leaves Protein Extraction

The protein in the leaves was extracted using the method described previously (Deyong et al., 2020; Zeng et al., 2020). The rice leaves were grounded into powder in liquid nitrogen, mixed with a trichloroacetic acid/ethanol mixture (1:9), centrifuged and the supernatant was removed. The pellet was air-dried after centrifugation. Then, STD buffer (4% SDS, 1 mM DTT, 150 mM Tris–HCl pH 8.0) was added to the precipitate. After sonication, it was placed in a boiling water bath and kept for 5 min. Next, the supernatant was centrifuged and the total protein content was calculated with bicinchoninic acid (BCA). Then, 200 μg of protein was taken and 200 μL of buffer (8 M Urea, 150 mM Tris–HCl pH 8.0) was added for protein digestion. The absorbance was measured at 280 nm to quantify the peptide.

### iTRAQ Labeling and Peptides Analysis

ITRAQ labeling and peptides analysis was performed according to the method we described earlier (Deyong et al., 2020; Zeng et al., 2020). Take 80 μg peptides from each group for iTRAQ labeling with iTRAQ Reagent-8plex Multiplex Kit (AB SCIEX, United States) according to the instructions. Subsequently, EASY-nLC 1,000 liquid chromatograph (Thermo Finnigan, United States) was used for separation, and Q-Exactive mass spectrometer (Thermo Finnigan) was used for identification. Then the raw data of the mass spectrometer was analyzed by the method described in Cui et al. (2019). The UniProt database (^1^iTRAQ labeling and peptides analysisaccessed on 16 August 2020) was used for protein identification. When accessed, the database contained 148,104 rice protein sequences [Oryza sativa subsp. japonica (Rice, 39947)]. False discovery rate (FDR) < 0.01. The peptide mass tolerance was ± 10 ppm and the fragment mass tolerance were 0.2 Da. In order to determine the relative difference in protein abundance, we defined proteins with a fold change rate ≥ 1.2 or ≤ 0.83 and *p* < 0.05 as differentially abundant proteins (DAPs).

### Extraction of Metabolites and Metabolomics Analysis

Same as previous research (Zeng et al., 2021). About 0.1 g of sample was grounded into powder in liquid nitrogen, methanol: acetonitrile: water (1 mL, 2:2:1, v/v/v) was added and mixed well. Ultrasonic extraction (100 W, 60 min, 4°C) was performed kept standing for 60 min at −20°C, and centrifuged for 20 min (4°C, 14,000 *g*). The centrifuged supernatant was then vacuumed dry. Then 100 μL acetonitrile: aqueous solution (1:1, v/v) was added to the dried sample, and the supernatant was collected by centrifugation for analysis. The sample was separated by ultra-high-performance liquid chromatography (Agilent 1290, United States). The column used for separation was a HILIC column with a column temperature of 25°C, a flow rate of 0.3 mL/min, and an injection volume of 2 μL. The mobile phase consisted of A: water + 25 mM ammonium acetate + 25 mM ammonia, B: acetonitrile. The separated samples enter the Triple TOF 6600 mass spectrometer (AB SCIEX) to be identified. Data acquisition was conducted in full-scan mode in combination with information-dependent acquisition mode. The parameters were set as follows: ion spray voltage, 5,500 V (+) and 5,500 V (−); ion source temperature, 600°C (+) and 600°C (−); collision energy, 35 ± 15 eV(−); curtain gas of 30 PSI; The original data was converted into. mzML format by ProteoWizard, and then the XCMS program was used for peak alignment, retention time correction and peak area extraction. XCMS software parameter settings were as follows: For peak picking, cent Wave m/z = 25 ppm, peak width = c (10, 60), prefilter = c (10, 100). For peak grouping, bw = 5, mz wid = 0.025, minfrac = 0.5 were used. Metabolite structure identification was carried out by accurate mass matching (< 25 ppm) and secondary spectrum matching. Reference material databases built by Dalian Institute of Chemical Physics and Shanghai Zhongke New Life Biotechnology Co., Ltd. The data was input into the software SIMCA-P 14.1 (Umetrics, Umea, Sweden) for pattern recognition, preprocessed by Pareto-scaling, and then subjected to multi-dimensional statistical analysis. Orthogonal PLS-DA (OPLS-DA) and metabolic pathway analysis both used MetaboAnalyst 4.0 software^2^. Principal component analysis (PCA) was performed by R software. The pheatmap package of R software was used for hierarchical cluster analysis.

### Bioinformatics Analysis

Gene Ontology and eggnog databases were used for functional annotation and classification of DAPs. Kyoto Encyclopedia of Genes and Genomes (KEGG)^3^ was used to analyze the major metabolic pathways of DAPs and DEMs. We believed that the GO terms and KEGG pathways were significantly enriched when *P*-value ≤ 0.05 (Yang et al., 2018). In addition, we used https://www.ricedata.cn/gene/ search for DAPs related to rice agronomic traits.

### Total RNA Extraction and Real-Time PCR

As mentioned in the previous study (Deyong et al., 2020; Zeng et al., 2020), the rice leaves were fully ground in liquid nitrogen, and then we used TaKaRa kit (9767) to extract total RNA, and denaturing agarose gel electrophoresis and Micro Drop (BIO-DL Co., Ltd., Shanghai, China) were used to evaluate the quality and concentration of total RNA. Next, TaKaRa kit (RR037A) was used to reverse transcribe total RNA into cDNA. SYBR Premix Ex Taq II [TaKaRa kit (820A)] was used to detect gene expression. Relative expression levels of genes were determined using a relative quantitative method (2^–Δ Δ *CT*^) (Cui et al., 2019). All primers were listed in Supplementary Table 1.

## Results

### The Morphological Changes of Rice Progeny Induced by Spaceflight

**FIGURE 1:**
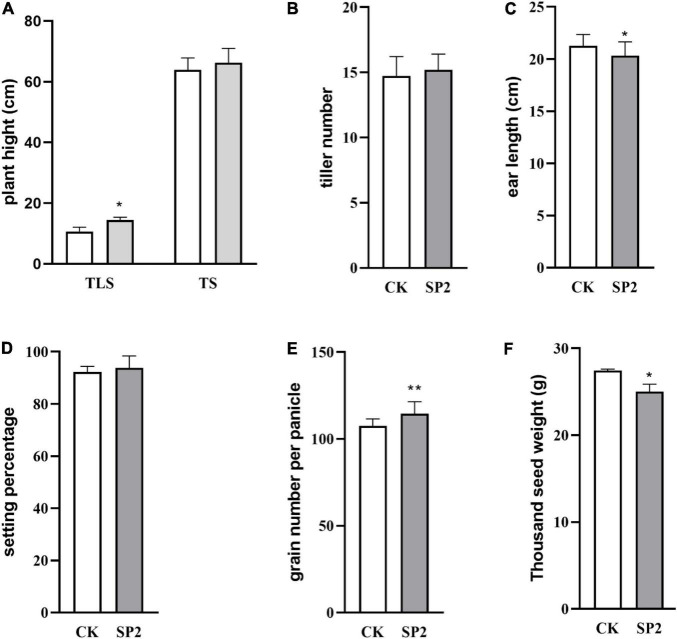
Change of plant height **(A)**, tiller number **(B)**, ear length **(C)**, setting percentage **(D)**, grain number per panicle **(E)**, thousand seed weight **(F)** in rice different stages of growth and development. (TLS, three-leaf stage; TS, tillering stage; White column represents the control group; Gray column represents treatment group). Data are mean ± SD, *n* = 30, * and ** indicate significant difference at *p* < 0.05 and *p* < 0.01 by student *t*-test, respectively.

In order to better understand the impact of spaceflight on rice progeny, we analyzed the agronomic traits of F2 plants at different growth and development stages (Figure 1). The plant height of the progeny after spaceflight increased significantly in TLS, while showed no significant difference in TS (Figure 1A and Supplementary Figure 1). Then we evaluated the impact of spaceflight on the tiller number ability of the F2 generation, which showed that the effective tiller number of the offspring did not change obviously after spaceflight (Figure 1B). Similarly, we evaluated the seed setting rate and ear length, and the results showed that there were rarely obvious changes in the F2 generation after spaceflight (Figures 1C,D). However, they showed significant differences in the number of grains without ears and thousand grain weight (Figures 1E,F). From the above results, we preliminarily concluded that the effect of spaceflight on rice still existed in the F2 generation of rice, which made the F2 generation plants of spaceflight show different agronomic traits from the control. This also showed that spaceflight had an impact on the physiological processes of F2 generation plants.

### The Physiological Changes of Rice Progeny Induced by Spaceflight

**FIGURE 2:**
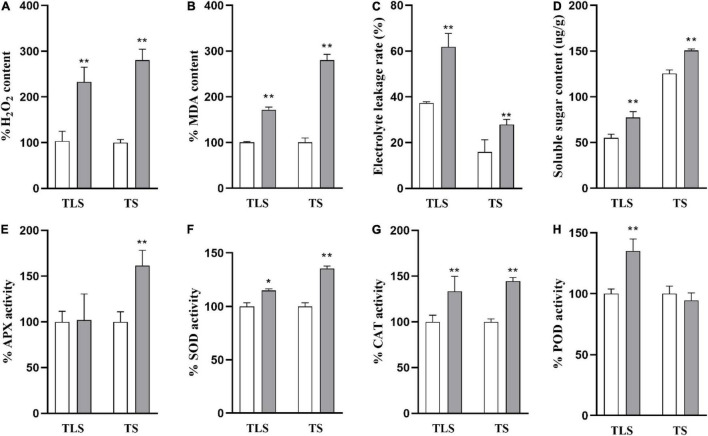
The physiological changes of rice induced by spaceflight. **(A)** Percent values of H_2_O_2_. **(B)** Percent values of MDA. **(C)** The soluble sugar contents. **(D)** The electrolyte leakage rate change. **(E)** Percent values of APX activity. **(F)** Percent values of SOD activity. **(G)** Percent values of CAT activity. **(H)** Percent values of POD activity (TLS, three-leaf stage; TS, tillering stage; White column represents the control group; Gray column represents treatment group. The data (mean ± SD) are the means of three replicates with standard errors shown by vertical bars, *n* = 3, * and ** indicate significant difference at *p* < 0.05 and <0.01 by student *t*-test, respectively.

In order to further explore the changes in the physiological processes of rice F2 generation plants after spaceflight, we measured the levels of H_2_O_2_, MDA, EL, SSC and the activity of antioxidant enzymes (Figure 2). In the TLS and TS, H_2_O_2_, MDA, EL, SSC in SP2 were much higher than that of the control (Figures 2A–D). In detail, H_2_O_2_ levels increased by 132.9 and 180.6%, respectively (Figure 2A), MDA levels increased by 71.4 and 179.7%, respectively (Figure 2B), EL levels increased by 66.2 and 79.1%, respectively (Figure 2C), SSC levels increased by 41.1 and 20.2%, respectively (Figure 2D), in TLS and TS. The SOD, POD and CAT activities all significantly increased by 14.9, 35.1, and 33.4%, respectively, in the TLS (Figures 2F–H). However, there was no significant difference in the activity of APX in the SP2 group compared to the control in the TS (Figure 2E). Similarly, our results showed that the activity of antioxidant enzymes changes during TS (Figures 2E,G,H). Totally, these results suggested that the redox balance of SP2 was broken.

### Protein Profiles of Rice Leaves at Different Developmental Stages

**FIGURE 3:**
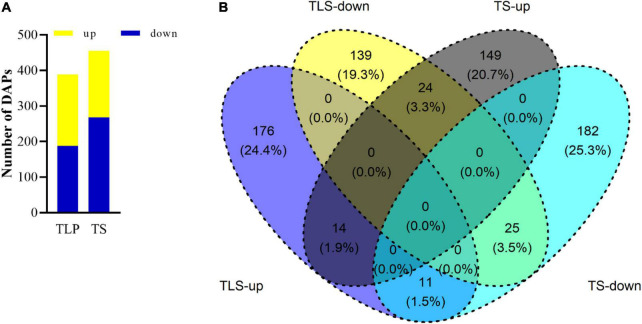
The number of up-regulated and down-regulated DAPs in two different growth stages **(A)**. Venn diagram showed the overlaps of the DAPs in in different growth stages **(B)**. TLS-up represented the three-leaf stage up-regulated proteins; TLS- down represented the three-leaf stage down-regulated proteins; TS-up represented tillering stage up-regulated proteins; TS-down represented tillering stage down-regulated proteins.

We used iTRAQ proteomics technology to evaluate the changes in the proteome during the two growth stages. A total of 3,867 proteins were identified in three biological replicates, and proteins with a ratio ≥ 1.2 (treatment group/control group) and adjusted *p* value were identified as differentially abundant proteins (DAPs). The abundances of 389 and 405 proteins were significantly changed in the TLS and the TS, respectively. Among them, 201 proteins were up-regulated while the other 188 proteins down-regulated in the TLS. Meanwhile, 187 proteins up-regulated and 268 proteins down-regulated during the TS (Figure 3A). The Venn diagram was used to characterize the overlap of proteins at different growth stages (Figure 3B). There were 315 and 331 unique proteins in TLS and TS, respectively. At the same time, there were 74 co-expressed proteins in the two growth stages. Among them, the expression abundance of 36 proteins showed opposite changes, and the expression trend of 38 proteins did not change (Figure 3B).

### Gene Ontology Annotation of DAPs

**FIGURE 4:**
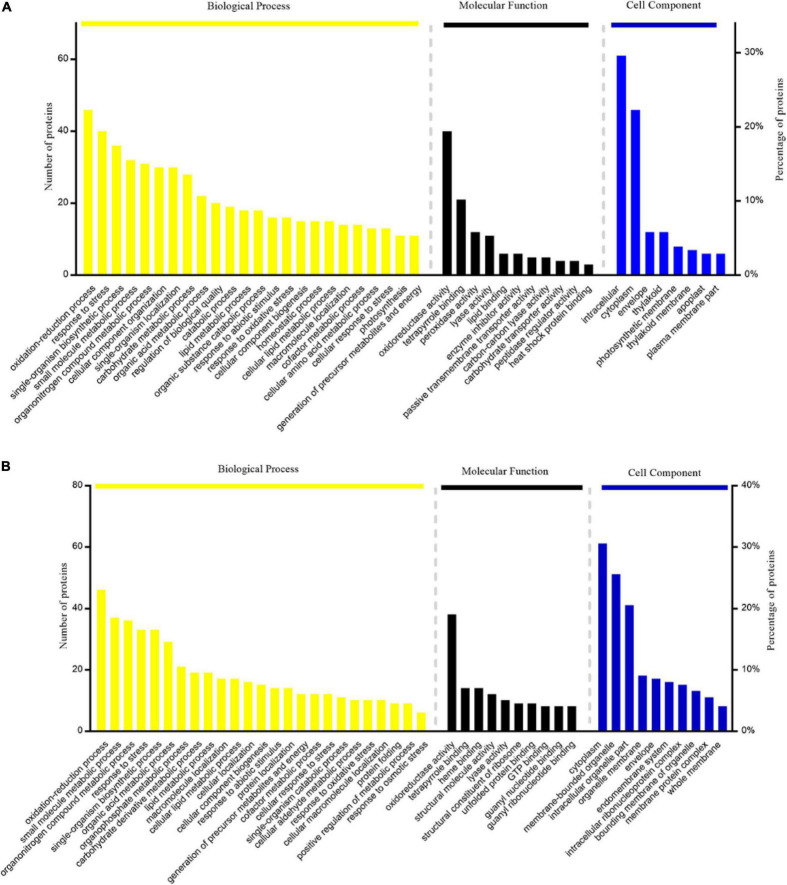
GO enrichment analysis of proteins with differential abundance: **(A)** three-leaf stage SP2 vs CK; **(B)** tillering stage SP2vs CK.

**FIGURE 5:**
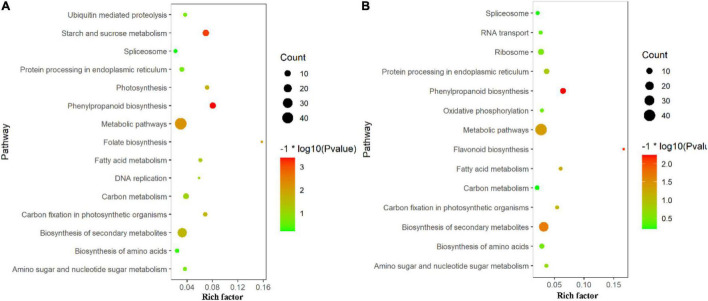
KEGG pathway enrichment analysis of proteins with differential abundance; TOP 15 of pathway enrichment; **(A)** three-leaf stage; **(B)** tillering stage.

In this study, Gene Ontology (GO) term enrichment was used to reveal the functional characteristics of DAPs. We selected GO items with level 4 for analysis. The biological processes (BP) that were significantly enriched in TLS include oxidation-reduction process, response to stress, single-organism biosynthetic process, small molecule metabolic process, etc. (Figure 4A). For molecular functions (MF) that were significantly enriched, there were oxidoreductase activity, tetrapyrrole binding, peroxidase activity, lyase activity, etc. (Figure 4A). The intracellular, cytoplasm, envelope, and thylakoid (Figure 4A) were the most abundant groups under Cell component (CC). Similarly, in the TS (Figure 4B), the BP analysis showed that oxidation-reduction process was the most representative term, followed by small molecule metabolic process, organonitrogen compound metabolic process, response to stress and single-organism biosynthetic process, etc. As for CC, cytoplasm, membrane-bounded organelle, intracellular part, etc. In the MF, the annotated proteins were mainly involved in the oxidoreductase activity, tetrapyrrole binding and heme binding, etc. In addition, we used the KEGG database to map DAPs on metabolic pathways. We have enriched 68 metabolic pathways in the 389 DAPs of TLS. They mainly included Phenylpropanoid biosynthesis, Starch and sucrose metabolism, Metabolic pathways, DNA replication, Biosynthesis of amino acids, Biosynthesis of secondary metabolites, Photosynthesis, etc. (Figure 5A). The 405 DAPs of TS were mapped to 67 metabolic pathways, including Phenylpropanoid biosynthesis, Flavonoid biosynthesis, Biosynthesis of secondary metabolites, Metabolic pathways, Biosynthesis of amino acids, Oxidative phosphorylation, etc. (Figure 5B).

### Functional Classification of DAPs

**FIGURE 6:**
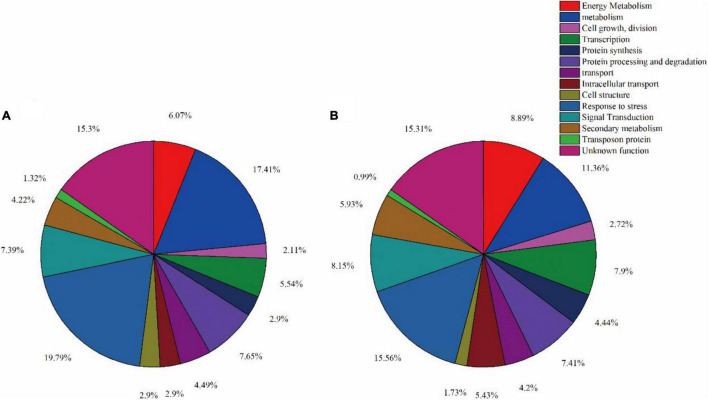
Function classification of differentially expressed proteins in leaves at three-leaf stage **(A)** and tillering stage **(B)**.

In order to better understand the impact of spaceflight on rice progeny, we classified the functions of DAPs according to the method described by Bevan et al. (1998) and the results of GO annotation. We divided the function of protein into 14 major functional categories and several functional sub-categories including metabolism, energy metabolism, cell growth/division, etc. (Supplementary Tables 2, 3 and Figure 2). The 389 DAPs in the TLS leaves were related to energy metabolism (6.07%), metabolism (17.41%), cell growth and division (2.11%), transcription (5.54%), protein synthesis (2.11%), protein processing and degradation (7.65%), transport (4.49%), intracellular transport (2.90%), cell structure (2.90%), response to stress (19.79%), signal transduction (7.39%), secondary metabolism (4.22%), transposon protein (1.32%) and unknown (15.30%) (Figure 6A). The 405 differentially expressed proteins in the TS leaves were related to energy metabolism (8.89%), metabolism (11.36%), cell growth and division (2.72%), transcription (7.9%), protein synthesis (4.44%), protein processing and degradation (7.41%), transport (4.20%), intracellular transport (5.43%), cell structure (1.73%), response to stress (15.56%), signal transduction (8.15%), secondary metabolism (5.93%), transposon protein (0.99%) and unknown (15.31%) (Figure 6B).

### Metabolome Profiles of Rice Leaves at Different Developmental Stages

LC-MS was used to evaluate the changes in the metabolic profile of rice offspring leaves by spaceflight. PCA was used to determine the degree of separation of the metabolic profiles of the SP2 group and the CK group. At the three-leaf stage, the difference between CK and SP2 was 75.91% (Supplementary Figure 2), and at the TS, the difference between CK and SP2 was 79.3% (Supplementary Figure 2). In the two periods, the CK group was completely separated from the SP2 group, which indicated that the impact of spaceflight on rice continued to the offspring. In the Supplementary Figure 2, all the data points formed close clusters between different comparison groups, which also reflected the repeatability of metabolomics results.

In order to find metabolites with significant differences, we performed an orthogonal PLS-DA (OPLS-DA) analysis (Supplementary Figure 3). We defined metabolites with a ratio ≥ 1.3, Variable of Importance to the Projection (VIP) > 1 and adjusted *p* value < 0.05 as differential metabolites (DEMs). A total of 124 DEMs were identified during TLS, of which 70 increased and 54 decreased (Supplementary Table 4). 125 DEMs were identified in TS, 87 of which increased and 38 decreased (Supplementary Table 5).

Hierarchical cluster analysis showed that the metabolites of SP2 and CK groups were separated from each other in two different growth and development stages (Supplementary Figure 4), which indicated that the impact of spaceflight panicle rice still existed in the offspring, and the physiological response of rice was still affected (Supplementary Figure 4). In addition, it indicated that the two growth and development stages of the F2 generation rice had different response mechanisms to the spaceflight (Supplementary Figure 4).

### Analysis of Metabolic Pathways of Rice Leaves at Different Developmental Stages

**FIGURE 7:**
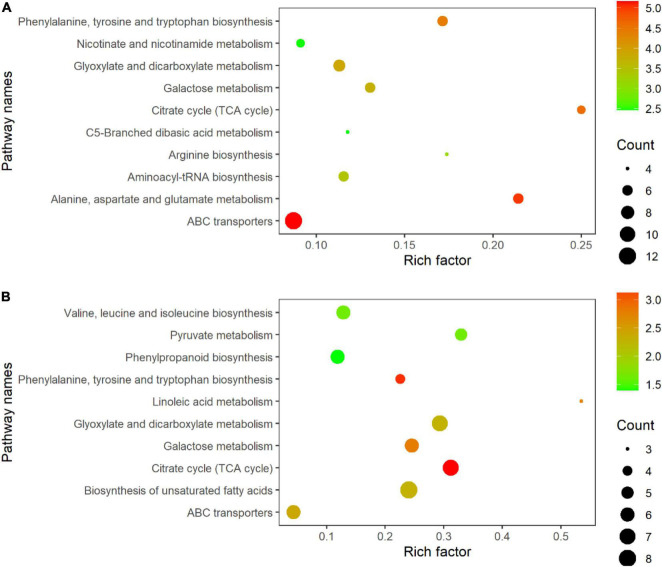
KEGG pathway enrichment analysis of metabolites with differential abundance; **(A)** three-leaf stage; **(B)** tillering stage.

All DEMs were submitted to the KEGG database for analysis of related pathways. The results showed that DEMs in TLS were mapped to 68 metabolic pathways (Supplementary Table 6), of which the top ten metabolic pathways mainly included ABC transporters; Alanine, aspartate and glutamate metabolism; Citrate cycle (TCA cycle); Phenylalanine, tyrosine and tryptophan biosynthesis; Glyoxylate and dicarboxylate metabolism; Galactose metabolism; Aminoacyl-tRNA biosynthesis; Arginine biosynthesis; Nicotinate and nicotinamide metabolism; C5-Branched dibasic acid metabolism (Figure 7A). Similarly, we also mapped the metabolic pathways of DEMs in TS, and a total of 66 pathways were enriched (Supplementary Table 7). The top ten pathways of these enriched metabolic pathways included citrate cycle (TCA cycle); Phenylalanine, tyrosine and tryptophan biosynthesis; Galactose metabolism; Linoleic acid metabolism; ABC transporters; Biosynthesis of unsaturated fatty acids; Glyoxylate and dicarboxylate metabolism; Valine, leucine and isoleucine biosynthesis; Pyruvate metabolism; Phenylpropanoid biosynthesis (Figure 7B). The changes in these metabolites and metabolic pathways provided important information on how the offspring of rice retained the memory of spaceflight stress.

### Comprehensive Analysis of Rice Leaves Metabolomics and Proteomics

In order to further understand the molecular mechanism of rice offspring in response to the spaceflight, we comprehensively analyzed the KEGG pathway of DAPs and DEMs. We found that the changes of DEMs and DAPs during TLS were related to 34 metabolic pathways (Table 1). Moreover, we found that there were 36 pathways that were changed by DAPs and DEMs in TS (Table 2).

**TABLE 1:** Proteins and metabolites involved in common pathways at TLS.

Pathway name	Metabolomics		Proteomics	
	Pathway ID	*P*-value	Pathway ID	*P*-value
Phenylalanine, tyrosine and tryptophan biosynthesis	map00400	3.6484E-05	osa00400	6.1700E-01
Glyoxylate and dicarboxylate metabolism	map00630	1.2823E-04	osa00630	1.6800E-01
Ascorbate and aldarate metabolism	map00053	1.2647E-02	osa00053	5.7100E-01
Glycine, serine and threonine metabolism	map00260	1.3556E-02	osa00260	7.2900E-01
Pyruvate metabolism	map00620	1.9406E-02	osa00620	8.2900E-01
Glutathione metabolism	map00480	3.3203E-02	osa00480	2.2500E-01
Cyanoamino acid metabolism	map00460	5.0944E-02	osa00460	4.2800E-02
Tyrosine metabolism	map00350	5.6997E-02	osa00350	5.8000E-01
Carbon fixation in photosynthetic organisms	map00710	6.7894E-02	osa00710	2.3200E-02
Fatty acid biosynthesis	map00061	9.3531E-02	osa00061	2.2700E-01
Purine metabolism	map00230	9.6256E-02	osa00230	9.4900E-01
Cysteine and methionine metabolism	map00270	1.1285E-01	osa00270	3.6700E-01
beta-Alanine metabolism	map00410	1.1973E-01	osa00410	5.1800E-01
Pentose phosphate pathway	map00030	1.3869E-01	osa00030	3.1900E-01
Biosynthesis of unsaturated fatty acids	map01040	1.6000E-01	osa01040	1.7700E-01
Monobactam biosynthesis	map00261	1.6489E-01	osa00261	2.2200E-01
Arginine and proline metabolism	map00330	1.7846E-01	osa00330	5.9900E-01
alpha-Linolenic acid metabolism	map00592	1.9873E-01	osa00592	2.1800E-01
Oxidative phosphorylation	map00190	2.6142E-01	osa00190	6.0100E-01
Fructose and mannose metabolism	map00051	2.6829E-01	osa00051	7.3500E-01
Linoleic acid metabolism	map00591	2.6991E-01	osa00591	2.8900E-01
Nitrogen metabolism	map00910	3.0232E-01	osa00910	4.7200E-01
Phenylpropanoid biosynthesis	map00940	3.6540E-01	osa00940	3.9400E-04
Vitamin B6 metabolism	map00750	4.1210E-01	osa00750	1.6600E-01
Glycolysis/Gluconeogenesis	map00010	4.4476E-01	osa00010	7.7100E-01
Thiamine metabolism	map00730	4.4476E-01	osa00730	2.0400E-01
Sulfur metabolism	map00920	4.6553E-01	osa00920	4.2800E-02
Starch and sucrose metabolism	map00500	5.0464E-01	osa00500	7.0900E-04
Amino sugar and nucleotide sugar metabolism	map00520	6.0660E-01	osa00520	2.3400E-01
Fatty acid degradation	map00071	6.1384E-01	osa00071	2.3500E-01
Pentose and glucuronate interconversions	map00040	6.4914E-01	osa00040	6.0800E-01
Biotin metabolism	map00780	7.5902E-01	osa00780	7.3100E-02
Ubiquinone and other terpenoid-quinone biosynthesis	map00130	8.2816E-01	osa00130	5.5000E-01
Porphyrin and chlorophyll metabolism	map00860	9.3529E-01	osa00860	5.7100E-01

**TABLE 2:** Proteins and metabolites involved in common pathways at TS.

Pathway name	Metabolomics		Proteomics	
	Pathway ID	*P*-value	Pathway ID	*P*-value
Citrate cycle (TCA cycle)	map00020	4.69E-04	osa00020	2.47E-01
Galactose metabolism	map00052	1.62E-03	osa00052	6.99E-01
Linoleic acid metabolism	map00591	1.72E-03	osa00591	4.25E-02
Biosynthesis of unsaturated fatty acids	map01040	5.11E-03	osa01040	1.74E-01
Glyoxylate and dicarboxylate metabolism	map00630	5.24E-03	osa00630	7.60E-01
Phenylpropanoid biosynthesis	map00940	4.03E-02	osa00940	5.81E-03
Glycolysis/Gluconeogenesis	map00010	4.55E-02	osa00010	5.19E-01
Pentose phosphate pathway	map00030	5.85E-02	osa00030	3.14E-01
Flavone and flavonol biosynthesis	map00944	5.99E-02	osa00944	6.52E-02
Cyanoamino acid metabolism	map00460	9.08E-02	osa00460	1.82E-01
Glycine, serine and threonine metabolism	map00260	1.09E-01	osa00260	3.63E-01
Glycerophospholipid metabolism	map00564	1.16E-01	osa00564	8.00E-01
Fructose and mannose metabolism	map00051	1.23E-01	osa00051	7.31E-01
Plant hormone signal transduction	map04075	1.28E-01	osa04075	9.86E-01
Phenylalanine metabolism	map00360	1.46E-01	osa00360	5.46E-01
Glutathione metabolism	map00480	1.52E-01	osa00480	2.20E-01
Flavonoid biosynthesis	map00941	2.03E-01	osa00941	6.80E-03
Riboflavin metabolism	map00740	2.04E-01	osa00740	1.83E-01
Sphingolipid metabolism	map00600	2.48E-01	osa00600	3.91E-01
Purine metabolism	map00230	2.91E-01	osa00230	9.48E-01
Lysine biosynthesis	map00300	3.29E-01	osa00300	2.87E-01
Starch and sucrose metabolism	map00500	3.44E-01	osa00500	6.90E-01
Amino sugar and nucleotide sugar metabolism	map00520	3.46E-01	osa00520	2.28E-01
alpha-Linolenic acid metabolism	map00592	3.51E-01	osa00592	5.46E-02
Ascorbate and aldarate metabolism	map00053	4.14E-01	osa00053	1.98E-01
Pentose and glucuronate interconversions	map00040	4.67E-01	osa00040	6.04E-01
Monobactam biosynthesis	map00261	4.77E-01	osa00261	2.19E-01
Fatty acid biosynthesis	map00061	4.85E-01	osa00061	2.23E-01
Carbon fixation in photosynthetic organisms	map00710	4.95E-01	osa00710	7.79E-02
Arachidonic acid metabolism	map00590	5.77E-01	osa00590	2.02E-01
Nitrogen metabolism	map00910	6.23E-01	osa00910	1.27E-01
Sulfur metabolism	map00920	7.05E-01	osa00920	5.46E-01
Porphyrin and chlorophyll metabolism	map00860	8.93E-01	osa00860	5.66E-01
Fatty acid degradation	map00071	9.52E-01	osa00071	6.04E-01
Cysteine and methionine metabolism	map00270	9.77E-01	osa00270	8.88E-01

**FIGURE 8:**
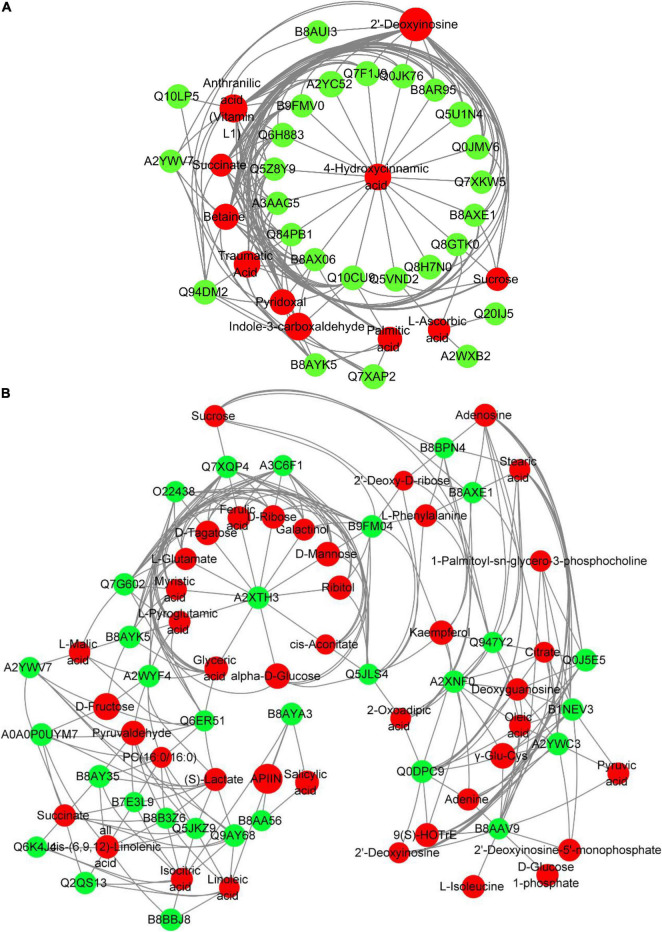
Network analysis of the mutual regulation relationship between different proteins and different metabolites in the same KEGG pathway. Red indicates differential metabolites, and green indicates DAPs. The size of the dots represents the difference multiples of different metabolites and DAPs. The greater the difference multiples, the larger the dots. **(A)** three-leaf stage; **(B)** tillering stage.

To further evaluate the interaction between DAPs and DEMs, we assessed the correlation of DAPs with DEMs by Pearson’s test and visualized the correlation network using cytoscape (Figure 8). In TLS, 4-Hydroxycinnamic acid was at the center of the entire network, and the changes in the abundance of 11 types of DAPs [Q84PB1 (phosphoribosyl anthranilate transferase), Q7F1J9 (L-ascorbate peroxidase), Q7XKW5 (L-threonine aldolase 1), B9FMV0 (Malic enzyme), Q0JK76 (Glutathione-*S*-transferase), Q10CU9 (Glycosyl hydrolase family 3 N terminal domain protein), Q8H7N0 (alcohol dehydrogenase), Q5VND2 (Cysteine synthase), Q5Z8Y9 (Cysteine synthase), B8AXE1 (3-hydroxyacyl-CoA dehydrogenase), B8AR95 (COX6B), Q6H883 [H(+)-exporting diphosphatase), Q0JMV6 (Os01g0357100 protein), A2YC52 (Peroxidase), Q5U1N4 (Class III peroxidase 59), Q8GTK0 (Starch synthase), A3AAG5 (Methyltransf_11 domain-containing protein), B8AX06 (UbiA prenyltransferase family)] were related to changes in the content of 4-Hydroxycinnamic acid (Figure 8A). Interestingly, among these 11 proteins, there are proteins involved in energy metabolism (COX6B, Malic enzyme), redox balance (Class III peroxidase 59, Peroxidase, L-ascorbate peroxidase), and sugar metabolism (glutathione Stransferase, Glycosyl hydrolase family 3 N terminal domain protein, Starch synthase). This may suggest that changes in these pathways may be one of the reasons for the changes in 4-Hydroxycinnamic acid content in rice during spaceflight-induced TLS. Further, in TS, the protein A2XTH3 (Peroxidase) was located in the center of the network, and there were 12 metabolites (Ferulic acid, L-Glutamate, Ribitol, L-Pyroglutamic acid, Myristic acid, alpha-D-Glucose, D-Mannose, D-Ribose, Glyceric acid, D-Tagatose, Galactinol, *cis*-Aconitate) content changes directly related to their abundance changes (Figure 8B). Most of these 12 metabolites were organic acids and sugars. Notably, changes in peroxidase abundance in both TLS and TS appear to be related (directly or indirectly) to glucose metabolism. Furthermore, our results show a reciprocal regulatory relationship between changes in DAPs and DEMs.

### Analysis of the Comprehensive Systemic Metabolic Pathways Diagram

**FIGURE 9:**
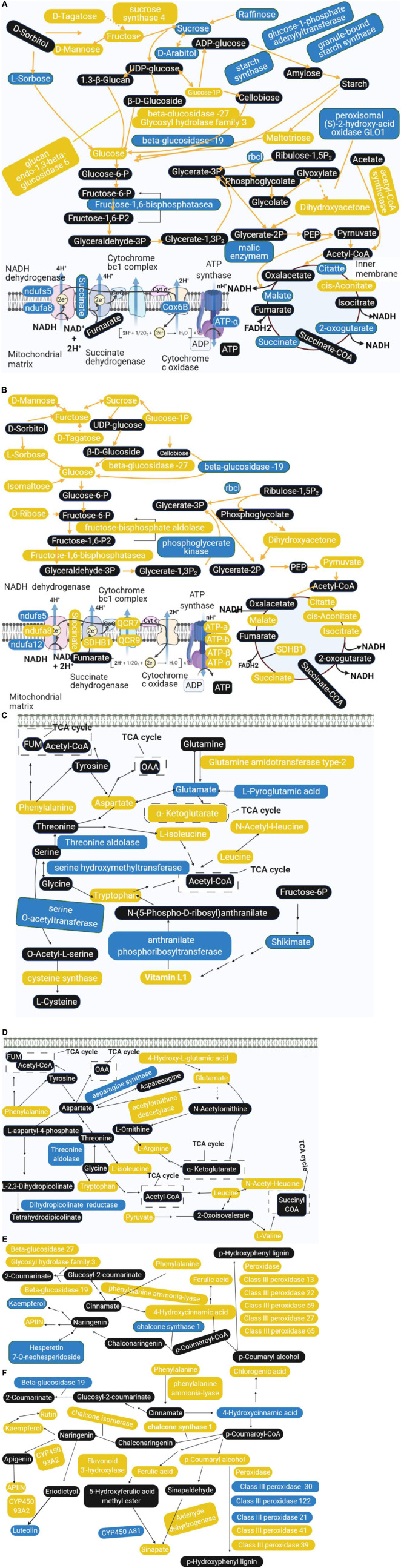
Main biological pathway responses to spaceflight stress in rice progeny. **(A)** Carbohydrate metabolism and energy metabolism in TLS; **(B)** carbohydrate metabolism and energy metabolism in TS; **(C)** amino acid metabolism in TLS; **(D)** amino acid metabolism in TS; **(E)** phenylpropane biosynthesis and flavonoid metabolism in TLS; **(F)** phenylpropane biosynthesis and flavonoid metabolism in TS. The DEPs and metabolites are marked in box; yellow indicates upregulation; blue indicates downregulation; black indicates no significant change.

Combining the results of proteomics and metabolomics, we found that the amino acid metabolism in the offspring rice was abnormal, accompanied by related metabolites and protein metabolism disorders. Sugar metabolism has been affected, and energy metabolism has also been disturbed. It is worth noting that the amino acid metabolism has also been affected. In addition, phenylpropane biosynthesis and flavonoid metabolism showed significant changes. Based on this, we constructed a comprehensive systemic metabolic pathway map to reveal the response mechanism of rice progeny plants to the spaceflight (Figure 9). In TLS, the abundance of a total of seven proteins changed significantly during the process of sugar metabolism. The abundance of sucrose synthase 4, glucan endo-1,3-beta-glucosidase 6, beta-glucosidase −27, and Glycosyl hydrolase family three increased, which may account for the accumulation of fructose and glucose. The content of intermediate metabolites during the TCA cycle in TLS was reduced, and the abundance of related proteins in the electron transport chain was reduced either, which may lead to a reduction in energy metabolism flux at this time (Figure 9A). Unlike TLS, only one glycometabolizing protein abundance changed in TS, and D-Mannose, fructose, Sucrose, D-Tagatose, L-Sorbose, Isomaltose, Glucose, and D-Ribose were all accumulated. The changes in the abundance of related metabolites and proteins in the TCA cycle and the electron transport chain were increased, which indicated that energy metabolism was activated compared to the control group (Figure 9B). There were six proteins involved in amino acid metabolism in TLS. In addition to the up-regulated expression of Glutamine amidotransferase type-2, the abundance of other proteins was down-regulated (Figure 9C). In TS, only four proteins were involved in amino acid metabolism at this time (Figure 9D). Obviously, phenylpropane biosynthesis and flavonoid metabolism in TS had a more complex regulatory network than in TLS (Figures 9E,F). Interestingly, chalcone synthase 1 may have an important influence on the content of Rutin, Kaempferol, APIIN and Hesperetin 7-*O*-neohesperidoside (Figures 9E,F).

### mRNA Expression Validations

**FIGURE 10:**
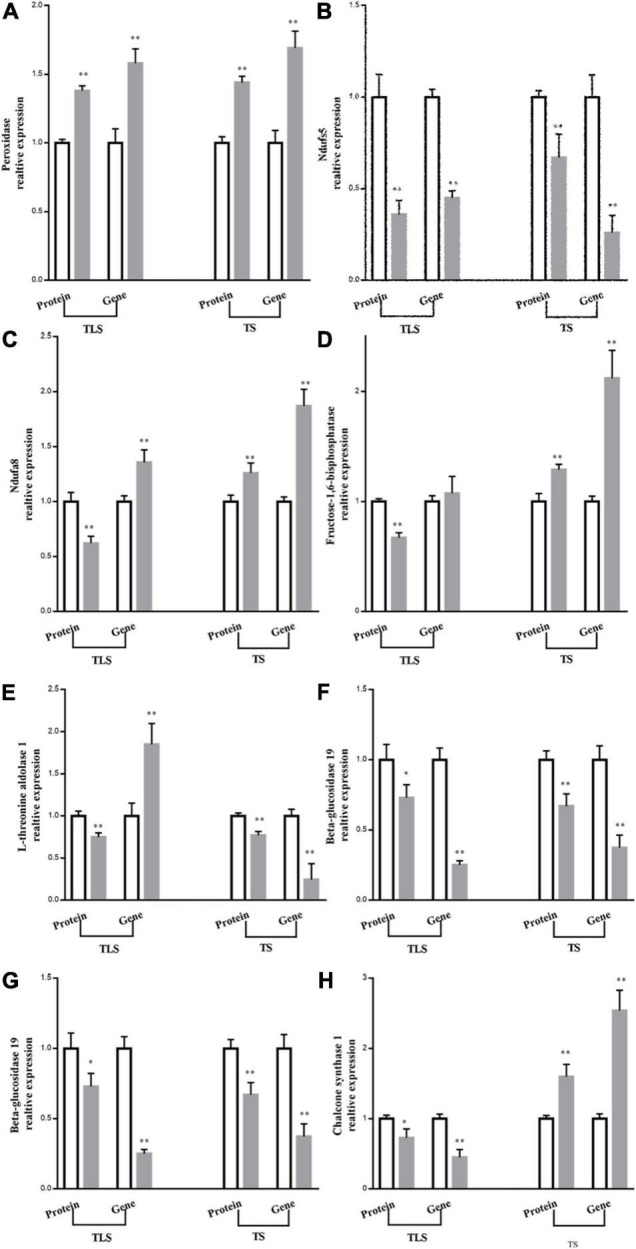
Comparison between protein abundance and mRNA level. The protein abundance in ground treatment and after spaceflight treatment leaves at three-leaf stage (TLS) and tillering stage (TS) were assessed by ITRAQ based quantitative proteome analysis. Transcript abundance at two development stages (TLS, TS) was determined by quantitative RT-PCR and normalized against the *ACTIN* gene. The white column represents the control group; gray column represents treatment group. The means and standard error values from three independent samples are shown (means ± SE; *n* = 3). * and ** indicate significant difference at *p* < 0.05 and <0.01 by student *t*-test, respectively.

qRT-PCR was used to further verify the validity of the results. This study selected eight proteins involved in amino acid metabolism, sugar metabolism, energy metabolism, and phenylpropane metabolism, and showed significant differences in TLS and TS for qRT-PCR (Figure 10). In TS, the expression abundance of the eight proteins was the same as the expression trend of the genes encoding them, which confirmed that the iTRAQ results was reliable (Figure 10). Moreover, in TLS, the protein expression trend of Ndfua8 and fructose-1,6-bisphosphatase was different from the gene expression trend (Figures 10C,D). Figure 6 showed that DAPs involved in transcription modification and protein synthesis were enriched. The process of gene transcription to protein synthesis was affected, which may also explain why gene and protein expression trends were not the same, which further indicated that mRNA expression levels had limited effects on proteins. Therefore, it is more scientific to use multi-omics to reveal the mechanism of plants adapting to abiotic stress.

## Discussion

Spaceflight is a special abiotic stress. We have reported that spaceflight causes ROS accumulation in contemporary rice plants to respond to the impact of spaceflight, thereby changing protein expression and metabolite changes to achieve metabolic rearrangement. Thus, it is necessary to further study whether the memory of spaceflight stress is retained in rice progeny plants after spaceflight, and how to adapt to the effects of spaceflight through metabolic rearrangement. In this study, we have used metabolomics and proteomics to systematically analyze how offspring rice responded to the effects of spaceflight stress.

### Effects of Spaceflight on Agronomic Characters of Offspring Rice

The results of this study showed that after space flight, the plant height of the F2 generation plants increased significantly at the TLS, but there was no significant change at the TS (Figure 1A), which has the same trend as the plant height of F1 generation rice plants after space flight (Deyong et al., 2020). Moreover, compared with the CK group, the SP2 group had significant changes in ear length, number of grains per ear, and 1,000-grain weight (Figures 1C,E,F), while the number of tillers and seed setting rate did not change significantly (Figures 1B,D). The agronomic traits of plants often change when they are subjected to abiotic stress (Bali and Sidhu, 2020). Under drought, salt stress, high temperature, and heavy metal stress, the plant height, ear length, grain and growth rate of crops would change (Saleem, 2003; [Bibr B56][Bibr B7]; Abbas, 2012), and it is believed that the impact of abiotic stress on agronomic traits is related to the accumulation of ROS (Thounaojam et al., 2012; Bali and Sidhu, 2020). Therefore, the changes in agronomic traits of the F2 generation plants suggested that the F2 generation plants still retained the effects of spaceflight stress. In order to further explore the reasons for the changes in the phenotype of the F2 generation plants, we compared all DAPs with the proteins that control the phenotype in the https://www.ricedata.cn/gene/database. We found that in TLS, two DAPs (Q5QM60, Q6YYV8) were associated with rice plant height, and their abundance both increased. Q5QM60 (photoperiod-sensitive dwarf 1) deficiency can lead to impaired cell division and elongation, and severely dwarf plants under long-day conditions, and neither gibberellin nor brassinosteroids can save plant height changes caused by this gene (Li et al., 2014). Q6YYV8 (BAHD acyltransferase-like protein gene; slender grain Dominant), maintained the steady state of brassinosteroids in the plant. Silencing this gene would result in smaller rice grains and dwarf plants, but over-expression of this gene would not change the existing phenotype (Feng et al., 2016). Therefore, in this study, the change in plant height during TLS was mainly due to the difference in the expression of photoperiod-sensitive dwarf 1. Although there was no significant change in plant height during TS, we also found two DAPs related to plant height. A0A0P0VS15 (basic transcription factor 3) was a basic transcription factor, and inhibiting its expression would result in rice plant dwarf and typical pollen abortion. In addition, basic transcription factor 3 was regulated by abiotic stresses such as salt, high temperature and exogenous plant hormones (Wang et al., 2012). Q6EUP4 (GA-insensitive dwarf 2), which was an F-box subunit of a SCF E3 complex, mediated GA signal transduction in rice and affects rice plant height. Rice mutants that silence GID2 exhibited severe dwarfing, broadened leaves, dark green in color, and sterility (Gomi et al., 2004). We found a significant change in the protein that controlled the number of grains per panicle (Q03200). Q03200 (Light-induced rice 1, LIR1) was a chloroplast protein, which regulated the adhesion of ferredoxin NADP^+^ oxidoreductase (LFNR) to thylakoid membrane. LIR1 and LFNR form thylakoid protein complexed with TIC62 and TROL. LIR1 can increase the affinity of LFNR and TIC62. Light induced the rapid degradation of LIR1 and releases LFNR from Thylakoid Membrane. Silencing the protein showed growth retardation, and the seeds produced were about 75% of the control group (Yang et al., 2016).

### The F2 Generation Rice Still Retained the Memory of Spaceflight Stress

Among the various types of ROS, H_2_O_2_ has received the most attention (Xie et al., 2019). Several recent studies have shown that H_2_O_2_ can activate multiple acclamatory responses that reinforce resistance to various abiotic stressors (Li et al., 2019; Hasanuzzaman et al., 2022). Excessive ROS can lead to oxidative stress (Hasanuzzaman et al., 2020). Oxidative stress is the driving force for evoking stress responses. MDA content is one of the cytotoxic chemicals that determine oxidative damage to cell membranes and the final product of lipid peroxidation (Hossen et al., 2022). Electrolyte leakage (EL) is a sign of stress response in plant cells, which is usually accompanied by the accumulation of reactive oxygen species (ROS) and often leads to programmed cell death (PCD) (Demidchik et al., 2014). Soluble sugar is considered to be an important molecule for sensing the concentration of ROS in cells, and involves in the response of plants to oxidative stress (Afzal et al., 2021). Studies have reported that H_2_O_2_, EL, and MDA increased in rice under salt stress. Salt stress increased EL in soybean by 69%, while H_2_O_2_ and MDA contents increased by 75 and 56%, respectively (Alharby et al., 2021). Similar results were observed in our study (Figure 2). In the SP2 group, these four oxidative stress markers all increased (Figures 2A–D), which indicated that the F2 generation plants still retained the stress response generated by space flight.

Antioxidative defense systems protect plants from oxidative damage under stress by detoxifying ROS and maintaining the balance of ROS production under abiotic stress (Xie et al., 2019). Antioxidative enzymes are an important part of plant antioxidant system. It was reported that the activities of CAT and APX in plants increased with the intensification of salt stress (Abulfaraj and Jalal, 2021). Meanwhile, salt stress can lead to the increase of SOD activity and APX activity in rice (Hossen et al., 2022). The activities of SOD, CAT, and APX were increased in soybean plants treated with 7.46 dS m^–1^ NaCl (Soliman et al., 2020). Soybean plants treated with 100 mM NaCl for 25 days increased SOD, CAT, and APX activities by 31, 16, and 20, respectively (Taha et al., 2020). In this study, we evaluated changes in the activities of four antioxidant enzymes. The results showed that the activities of the four antioxidant enzymes in the SP2 group changed during TLS and TS, but there were differences in the changes in enzyme activities during the two periods (Figures 2E–H), which indicated that there may be differences in their ways to eliminate ROS. This also shows that oxidative stress exists in SP2 group again.

Searched for DAPs related to ROS clearance (Table 3). In TLS, 13 proteins related to ROS clearance were differentially expressed, and most of them were Peroxidase family proteins. It is worth noting that the expression abundance of these proteins increased, indicating that the redox state of the SP2 group plants was severely damaged at this time. In TS, there were nine proteins that were related to ROS clearance. Two of these nine proteins were Glutathione S-transferase family proteins, and the rest were Peroxidase family proteins. We noticed that the enzyme activities of APX and CAT have changed, while the protein abundance has not changed significantly, which indicated that the changes in the activity of these two enzymes may not be affected by the protein abundance. The changes in the abundance of these proteins further indicated that the stress response caused by space flight was still retained in the F2 generation of plants, and continued to the F2 generation of TS. There were also some flavonoids accumulated in this study, which would be analyzed in the follow-up discussion.

**TABLE 3:** Changes in protein abundance associated with ROS scavenging in the three-leaf stage (TLS) and the tillering stage (TS) as identified by iTRAQ.

Uniprot ID	Description	Fold change		Adjusted *P* value	
		TLS	TS	TLS	TS
A2YC52	Peroxidase	2.31		2.2903E-03	
Q5U1T0	Class III peroxidase 13	1.88		9.2736E-04	
Q5U1N4	Class III peroxidase 59	1.88		3.2026E-04	
Q7F1J9	L-ascorbate peroxidase	1.6		3.5152E-03	
A2WNR8	Peroxidase	1.51		6.1473E-05	
A0A0P0XR31	Peroxidase	1.46		2.8026E-02	
Q94DM2	Class III peroxidase 22	1.42		1.0709E-02	
Q6AVZ8	Class III peroxidase 65	1.41		3.6169E-02	
A2XTH3	Peroxidase	1.38	1.44	4.0609E-02	2.2156E-03
Q9ST82	Peroxidase	1.35		3.9481E-02	
Q6EUS1	Class III peroxidase 27	1.32		4.0609E-02	
Q0JK76	Glutathione Stransferase	1.32		4.0609E-02	
A2XGP6	Superoxide13 dismutase (Cu-Zn) 1	1.27		7.6207E-04	
B1NEV3	Peroxidase		1.3		1.0556E-03
Q5U1Q2	Class III peroxidase 41		1.26		1.0564E-02
Q5U1Q4	Class III peroxidase 39		1.25		4.1361E-03
Q6K4J4	Class III peroxidase 122		0.76		6.3815E-03
O22438	Class III peroxidase 21		0.73		1.0191E-02
Q93WY5	Glutathione S-transferase		0.72		9.7683E-03
Q6ER51	Class III peroxidase 30		0.68		4.5544E-03
Q6QN17	Glutathione S-transferase GSTU35		0.34		7.1210E-06

*Blue represents down-regulated.*

*Yellow represents up-regulated.*

What’s interesting is why the F2 generation plants can retain the stress response caused by space flight? It is well known that the most serious damage to the body caused by space flight was DNA damage (Moreno-Villanueva et al., 2017), the genomic instability caused by which can lead to heritable environmental stress (Boyko and Kovalchuk, 2011). Therefore, we speculated that the stress response of F2 generation plants to the space environment was caused by DNA damage and genome instability. To confirm this conjecture, we explored the changes in proteins and metabolites related to DNA damage and repair (Table 4). In TLS, there were three different proteins involved in the DNA repair process. Q852K3 (Replication factor C subunit 5) was a subunit of Replication factor C complex, which was involved in DNA repair, DNA replication and checkpoint control in cell cycle progression. The biological function of ScRFC5 in rice DNA repair process was still unclear, but it was only necessary for embryo development and mitosis at the cell stage (Chen et al., 2018). However, in yeast cells, ScRFC5 was necessary for DNA damage checkpoint control (Naiki et al., 2000). The lack of B8BHL4 (DNA ligase) increased the sensitivity of plants to ionizing radiation, and it participated in T-DNA integration and regulation mechanisms in DNA repair (Friesner and Britt, 2003). B8AKX8 (Cullin 4) was an important member of the Cullin family. It acted as a scaffold in the CUL4-DDB1-based ubiquitin ligase and regulated cell proliferation, DNA repair and genome integrity through key regulatory factors of ubiquitination (Lee and Zhou, 2007). Interestingly, the expression abundance of these three proteins related to DNA damage repair was all down-regulated in the TLS in the SP2 group, indicating that the DNA damage response existed in the plant and may affect the development of the plant simultaneously. In TS, we found two proteins and one metabolite related to DNA damage repair in the SP2 group. Q9FTU2 (replication factor A1) is an important protein for double-strand break repair in the process of meiotic homologous recombination (Liu et al., 2013), and help mediate genome stability and transcriptional gene silencing (Liu et al., 2010). Q0J8Y6 (DNA mismatch repair protein MutS2) is a member of the MutS family of proteins. This family of proteins participate in the process of DNA mismatch repair and is a natural candidate for maintaining a low mutation rate in the plant cell genome (Wu et al., 2020). Generally, deoxyguanosine can effectively inhibit the division of plant cells (Brulfert et al., 1974), which can be transformed into a DNA damage marker 8-oxo-2′-deoxyguanosine *in vivo*. Therefore, the increase in the content of the compound can reflect the damage of DNA. We noticed that the abundance of proteins involved in the DNA repair process in the SP2 group of plants decreased during TLS and TS, which was unfavorable for maintaining the genomic stability of the dimensional plant, indicating that the space flight reduced the genomic stability of the F2 generation plant and retained the stress response brought about by the space flight.

**TABLE 4:** Differentially expressed proteins and metabolites related to DNA damage.

Uniprot ID	Description	Fold change		Adjusted *P* value	
		TLS	TS	TLS	TS
**Protein**					
B8BHL4	DNA ligase	0.76		4.0332E-02	
Q852K3	Replication factor C subunit 5	0.71		2.2429E-02	
B8AKX8	Cullin 4	0.44		2.5086E-04	
Q9FTU2	Replication factor A1		0.77		1.0564E-02
Q0J8Y6	DNA mismatch repair protein MutS2		0.75		1.9912E-04
**Metabolites**					
	Deoxyguanosine		1.32		1.1766E-02

*Blue represents down-regulated.*

*Yellow represents up-regulated.*

### Saccharide Metabolism Was Involved in the Response of F2 Generation Plants to the Spaceflight

Sugar plays a central role in maintaining plant cell structure and metabolism, and participates in the response of plant cells to abiotic stress (Kumari and Parida, 2018). Under different abiotic stresses, there are almost no consensus on the changes of specific carbohydrates in different species, which implies that there are different metabolic rearrangements in different species (Kumari and Parida, 2018). In addition, sugar can also act as a signal molecule for nutrients and metabolites in plant cells, and activate or interact with specific plant hormone transmission pathways, leading to changes in gene expression and protein abundance (Couée et al., 2006).

Monosaccharides are the basic structural unit of carbohydrate molecules and an important part of soluble sugars in plant cells. Compared with CK, the content of D-glucose in the SP2 group increased approximately 2.27 times and 2.93 times during the TLS and TS stages, respectively (Table 5). The accumulation of D-glucose can reduce the damage of salt stress to wheat seedlings and improve its photosynthetic capacity (Wang et al., 2019). D-Fructose was accumulated 3.06 and 3.70 times in the TLS and TS stages, respectively (Table 5). Fructose participated in the antioxidant protection of plants with a high ability to remove ROS, which was twice that of glucose (Bogdanović et al., 2008). At the same time, studies have shown that the accumulation of fructose under abiotic stress was also related to the synthesis of erythrose-4-P, which was a substrate for the synthesis of lignin and phenolic compounds (Hilal et al., 2007). Moreover, the D-Tagatose and D-Mannose were also accumulated in the TLS and TS stages (Table 5). Studies have shown that D-Tagatose can inhibit the metabolism of D-Mannose (Mochizuki et al., 2020), and both sugars responded to abiotic stresses of plants (Shahbazy et al., 2020, (Nishizawa et al., 2008; Li et al., 2017; Kumari and Parida, 2018). D-Mannose reduced the damage under abiotic stress by removing ROS in plants (Nishizawa et al., 2008). This study has also found changes in other monosaccharides, such as L-Sorbose and D-Ribose (Table 5), which only changed during the TLS and TS stages, respectively.

**TABLE 5:** Differentially expressed proteins and metabolites related to saccharide metabolism.

Uniprot ID	Description	Fold change		Adjusted *P* value	
		TLS	TS	TLS	TS
**Protein**					
Q10CU9	Glycosyl hydrolase family 3 protein	1.41		1.2836E-03	
Q10LP5	Sucrose synthase 4	1.33		3.4614E-02	
Q84YK7	Beta-glucosidase 27	1.3		3.0213E-02	
A2YSI0	Glucan endo-1,3-beta-D-glucosidase	1.3		4.0609E-02	
D0TZC9	Glucose-1-phosphate adenylyltransferase	0.74		3.3343E-02	
Q8GTK0	Starch synthase	0.63		3.4245E-02	
A2Y7W1	Glucose-1-phosphate adenylyltransferase	0.58		2.2508E-03	
D0U0Q5	Soluble starch synthase II-3	0.5		7.6898E-04	
B8AXU3	Beta-glucosidase 19	0.73	0.67	2.6617E-02	1.0564E-02
**Metabolites**					
	D-Arabitol	4.34		1.6250E-05	
	D-Fructose	3.06	3.7	6.4945E-06	1.6293E-05
	Alpha-D-Glucose	2.27	2.93	1.6199E-06	7.6749E-04
	D-Tagatose	2.16	2.41	3.4048E-07	2.5078E-03
	D-Mannose	2.1	2.47	1.9112E-06	1.1283E-05
	Maltotriose	2.05		6.1085E-04	
	Isomaltose		1.5		5.5054E-05
	D-Ribose		1.9		1.4766E-03
	D-Glucose 1-phosphate	1.711	1.25	1.0986E-03	2.4599E-04
	Sucrose	0.69	1.31	1.4117E-06	4.4672E-06
	L-Sorbose	0.69		1.8522E-07	
	Raffinose	0.39		4.0648E-05	

*Blue represents down-regulated.*

*Yellow represents up-regulated.*

In addition to monosaccharides, soluble sugars also included disaccharides (sucrose, trehalose), raffinose family oligosaccharides (RFO) and fructan, which were mainly involved in stress responses in plants (Keunen et al., 2013). Sucrose is the main product of plant photosynthesis and the basic form of sugar storage in plants (Hilal et al., 2007; Rosa et al., 2009), which is composed of glucose and fructose. Studies have shown that sucrose was closely related to plant growth, development, signal transmission and adversity adaptation (Hilal et al., 2007; Chen et al., 2019). The sucrose content in the SP2 group decreased by 31% during TLS, while increased by 1.31 times during TS, which may be due to the different hydrolysis rates of sucrose in different growth and development stages. Certainly, this also implied that there were differences in rice energy metabolism between the two growth and development stages. The results of this study also showed that the content of Raffinose and Maltotriose changed significantly during the TLS phase (Table 5). Mannose and raffinose can protect plant cells from oxidative damage caused by various stress conditions, which has been determined that they have the ability to eliminate ROS (Nishizawa et al., 2008).

As a major polysaccharide, starch is the most important supplier of various carbohydrates in plants. It regulates plant growth by producing energy through respiratory metabolism, and can be hydrolyzed into soluble sugars by amylase (Chen et al., 2019). Previous studies have shown that space flight affects plant starch metabolism (Guisinger and Kiss, 1999). In this study, three types (Soluble starch synthase II-3, Starch synthase, and Glucose-1-phosphate adenylyl transferase) were involved in the reduction of protein expression in starch synthesis (Table 5), which indicated the process of starch synthesis in TLS Be suppressed. In addition, Soluble starch synthase II-3 determines the structure type of starch in the process of starch synthesis. The absence of Soluble starch synthase II-3 would make the starch granules smaller and rounder. Therefore, our results suggested that in F2 generation plants, there may be difference in starch structure between the spaceflight group and the control group. The above results also suggested that in the SP2 group, plants may adjust sugar metabolism to eliminate excess ROS, and may induce the reconstruction of other metabolic pathways through sugar metabolism to adapt to the effects of spaceflight.

### Energy Metabolism Was Involved in the Response of F2 Generation Plants to the Spaceflight

Energy metabolism is one of the most important regulators for plants to adapt to abiotic stress (Gharechahi et al., 2015). We previously reported changes in the expression abundance of proteins involved in energy metabolism in contemporary rice plants after space flight (Deyong et al., 2020). Here we examined the changes in related proteins and metabolites during glycolysis, TCA cycle, and oxidative phosphorylation (Table 6). It has been proposed that glycolysis played an important role in the response of plants to abiotic stress. Three proteins involved in glycolysis were differentially abundant in the SP2 group. The expression abundance of Fructose-1,6-bisphosphatase changed significantly in both periods. Studies have shown that Fructose-1,6-bisphosphatase can regulate plant tolerance to abiotic stress (Chee Hark et al., 1997). Inhibiting the expression of this protein would result in reduced plant growth, dwarf phenotype and delayed flowering, as well as changes in important metabolites such as amino acids, sugars and organic acids (Rojas-González et al., 2015). Therefore, the changes in amino acid metabolism and sugar metabolism in this study may be related to the differential expression of the protein. Fructose-bisphosphate aldolase was involved in the production of D-glyceraldehyde 3-phosphate during glycolysis. And the expression of this protein was induced by salt, drought, heat and other abiotic stresses, and affects plant soluble sugar content, stem straightness, dry weight and seed size (Cai et al., 2018). In plants, Phosphoglycerate kinase converts 1,3-bisphosphoglycerate to 3-phosphoglycerate during glycolysis, and the reduction in the expression of this enzyme would also lead to reduced plant growth, photosynthetic capacity and starch content (Rosa-Téllez et al., 2018). In fact, these three enzymes participated not only in the glycolysis process, but also in the photosynthetic Calvin-Benson cycle reaction, thus having an impact on photosynthesis (Rojas-González et al., 2015; Cai et al., 2018; Rosa-Téllez et al., 2018).

**TABLE 6:** Differentially expressed proteins and metabolites related to energy metabolism.

Uniprot ID	Description	Fold change		Adjusted *P*-value	
		TLS	TS	TLS	TS
**Protein**					
Q7 × 8V5	Acetyl-coenzyme A synthetase	1.27		3.9493E-02	
P0C2Z4	ATP synthase subunit alpha	0.77		4.0609E-02	
B8AR95	Cytochrome c oxidase subunit 6b	0.65		1.6451E-02	
B8AY35	Fructose-bisphosphate aldolase		1.59		3.6623E-03
B9FW35	QCR7		1.51		1.2327E-03
Q5ZAS4	ATPase beta subunit		1.43		3.3091E-03
Q0DIC9	QCR9		1.31		3.4060E-03
A2WXB2	Fructose-1,6-bisphosphatase	0.67	1.29	2.0885E-02	3.2994E-03
A6N0U5	Ndufa8	0.62	1.26	7.3520E-03	1.0564E-02
Q75HQ5	ATP synthase subunit beta		1.26		6.3815E-03
A2XF65	Atpb		1.26		9.8779E-03
P0C2Y5	ATP synthase subunit a		1.25		1.0564E-02
Q9S827	SDHB1		0.79		1.0564E-02
Q7XBT1	NDUFA12		0.69		2.0238E-04
B8AAV9	Phosphoglycerate kinase		0.68		5.3781E-03
Q6ZJ19	Ndufs5	0.36	0.67	2.7798E-04	4.1503E-04
B7EBN1	Mitochondrial carrier protein		0.49		9.7310E-04
Q9FP98	TOM7-1		0.11		2.2796E-07
**Metabolites**					
	*cis*-Aconitate	1.71	5.35	3.2585E-04	4.5697E-10
	Citrate		1.76		2.1810E-06
	Pyruvic acid		1.55		6.1532E-07
	Isocitrate acid		1.23		4.8333E-02
	Succinate	0.58	1.3	1.0859E-07	4.3228E-02
	Alpha-ketoglutarate	0.44		4.7704E-07	
	L-Malic acid	0.15	2.09	7.5190E-10	6.7041E-04

*Blue represents down-regulated.*

*Yellow represents up-regulated.*

Recently, the TCA cycle is considered as a pressure sensor for plants, and changing the TCA cycle has been an inevitable response of plants under abiotic stress (Das et al., 2019). Under abiotic stress conditions, the TCA cycle was an important protective system (Fernie et al., 2004), and the increase in the TCA cycle helped to improve plant tolerance to abiotic stress (Zhong et al., 2016). In TLS, the SP2 group detected significant changes in four metabolites involved in the TCA cycle. Except for *cis-*Aconitate, the content of the other three metabolites decreased, indicating that the TCA cycle was inhibited at this time. However, during TS, there were six metabolites involved in the TCA cycle in the SP2 group, and the content of these six metabolites all increased, which showed that the TCA cycle was activated. These results suggested that the F2 generation plants have different responses to space flight stress at different growth stages. Many abiotic stresses led to the destruction of the mitochondrial electron transfer chain, which led to the reduction of ATP and the production of ROS.

It is well known that the reducing equivalent produced by the activity of the TCA cycle was used by the mitochondrial electron transport chain to promote the synthesis of ATP (Fernie et al., 2004). This study revealed that some proteins participated in the ETC (Table 6), NDUFS5, NDUFA12, NDFUA8, Cytochrome b-c1 complex subunit 9 (QCR9), Cytochrome b-c1 complex subunit 7 (QCR7), ATP synthase subunit beta, ATP synthase subunit alpha and ATP b. Our previous research has reported that NDUFS5 and NDUFA12 changed significantly in the F1 generation after spaceflight (Deyong et al., 2020). NDUFS5 and NDUFS12 have no contribution to the activity of complex I, but were related to their electron transfer efficiency (Loeffen et al., 1999). Obviously, the spaceflight caused a decrease in the electron transfer efficiency in rice mitochondrial complex I, which this effect continued from the F1 generation to the F2 generation.

QCR9, QCR7, TOM7-1, Mitochondrial carrier protein were necessary during the assembly process of mitochondrial complex III. The decrease in protein abundance of TOM7-1, Mitochondrial carrier protein would cause the assembly engineering of mitochondrial complex III to be hindered (Deyong et al., 2020). The changes of QCR9, QCR7, TOM7-1, and Mitochondrial carrier protein were also detected in the F1 generation plants after spaceflight (Deyong et al., 2020). This indicated that plant mitochondrial complex III may be a more sensitive part of space flight, and complex III was also one of the main sources of ROS. Therefore, the increase in rice ROS caused by space flight may be due to the dysfunction of complex III. Furthermore, we found that the expression abundance of ATP synthase changes. The abundance of mitochondrial electron transport chain-related proteins in the SP2 group decreased in TLS while increased in TS, which indicated that the rate of energy metabolism decreased in TLS and the respiratory rate increased in TS.

### Amino Acid Metabolism Was Involved in the Response of F2 Generation Plants to the Spaceflight

Previous studies have shown that the abundance of proteins related to amino acid metabolism in F2 generation plants changed after spaceflight (Ma et al., 2007; Wang et al., 2008). In higher plants, amino acids accumulated in response to various stresses and had multiple functions in plant growth (Less and Galili, 2008). Moreover, amino acids were also necessary for protein synthesis and provided necessary intermediate products for many metabolic reactions (Pratelli and Pilot, 2014).

In TLS, five types of amino acids were accumulated and 3 type of amino acids were reduced in SP2 group, and some proteins involved in amino acid metabolism also changed significantly (Table 7). In TS, seven types of amino acids were accumulated and one type of amino acids was reduced in SP2 group. Moreover, seven proteins involved in amino acid metabolism were altered in abundance during TS (Table 7). What’s interesting was that our results showed that the changes in amino acid metabolism-related proteins were inconsistent with the changes in amino acid content. Therefore, it can be speculated that the accumulation of amino acids in our study was caused protein hydrolysis instead of biosynthesis (Fan et al., 2018). In addition, plants can also activate the accumulation of amino acids in response to abiotic stress through sucrose signals (Jia et al., 2019). L-aspartic acid, leucine, isoleucine, valine, glutamic acid, and phenylalanine can generate TCA cycle intermediate products through oxidation. At the same time, the electrons generated during the oxidation process are directly sent to ETC. This would help the production of ATP under abiotic stress (Galili, 2011; Hildebrandt et al., 2015). In SP2, the changes in the content of these amino acids may be related to their participation in energy metabolism (Table 7). Moreover, these amino acids may also act as potential signal molecules themselves, or act as precursors for the synthesis of other secondary metabolites and plant hormones to trigger abiotic stress signals. For example, phenylalanine (Figure 9) can be used as a precursor of plant synthetic alkaloids and flavonoids (ROS scavengers) (Kumari and Parida, 2018). Therefore, the accumulation of phenylalanine can ensure the production of antioxidants in SP2 plants to improve their adaptation to spaceflight. Glutamate can be converted into alpha-ketoglutarate. In F2 plants, alpha-ketoglutarate was increased in TLS, while Glutamate content was reduced, which indicated that the increase in alpha-ketoglutarate content in SP2 plants was caused by the degradation of glutamate.

**TABLE 7:** Differentially expressed proteins and metabolites related to amino acid metabolism.

Uniprot ID	Description	Fold change		Adjusted *P*-value	
		TLS	TS	TLS	TS
**Protein**					
Q8H7N0	Alcohol dehydrogenase	1.44		1.0709E-02	
Q6K7D6	Lysine-ketoglutarate reductase	1.37		6.2624E-03	
Q01IY5	Amine oxidase	1.32		1.6010E-02	
Q5VND2	Cysteine synthase	1.32		4.0609E-02	
H9N066	Betaine aldehyde dehydrogenase	1.3		3.0213E-02	
A0A0P0YAL3	Phenylalanine ammonia-lyase	1.3		4.0609E-02	
A3C0A7	Sarcosine oxidase	1.29		4.0609E-02	
B9FLJ1	Glutamine amido transferase type-2	1.26		4.0609E-02	
Q0INQ6	Serine hydroxymethyl transferase	0.78		1.4128E-02	
B9FFK4	Anthranilate synthase component	0.78		4.0609E-02	
B9FHA5	Serine *O*-acetyltransferase	0.77		4.0609E-02	
Q84PB1	Phosphoribosyl anthranilate transferase	0.77		9.2369E-05	
Q6K6Q1	Phenylalanine ammonia-lyase		1.35		5.9680E-03
Q0JFF8	Hydroxy pyruvate reductase HPR3		1.27		4.8507E-03
B8AGS8	Acetylornithine deacetylase		1.27		3.2391E-03
A0A0P0W7B4	Amidase At4g34880		1.26		9.8779E-03
B9G099	Indole-3-glycerol phosphate synthase		0.79		3.3091E-03
Q7XKW5	L-threonine aldolase 1	0.75	0.77	1.1910E-02	5.6268E-03
A0A0P0WFZ1	Asparaginase		0.76		6.8962E-03
B7E3L9	Dihydropicolinate reductase		0.28		2.3522E-05
**Metabolites**					
	Leucine	3.3		3.7182E-06	
	Tryptophan	2.65		9.9516E-08	
	L-isoleucine	2.3	1.78	1.0986E-03	7.7095E-05
	Phenylalanine	2.08	1.49	2.0535E-05	2.0198E-04
	*N*-Acetyl-L-leucine	1.24	3.2	6.7834E-04	7.2787E-05
	L-Valine		2.14		4.7120E-02
	L-Arginine		1.95		4.1766E-02
	4-Hydroxy-L-glutamic acid		1.86		2.0251E-02
	Glutamate	0.37	1.29	1.0079E-07	1.0862E-02
	L-Pyroglutamic acid	0.69	0.63	5.6325E-06	1.5291E-04
	Aspartate	0.36		3.25E-06	

*Blue represents down-regulated.*

*Yellow represents up-regulated.*

### Phenylpropanoid Biosynthesis Pathway and Flavonoid Synthesis Metabolism Was Involved in the Response of F2 Generation Plants to the Spaceflight

It is well known that phenylpropane metabolism is the upstream reaction of flavonoid and lignin biosynthesis, and phenylalanine is an important precursor of phenylpropane metabolism (Cramer et al., 2013). In this study, phenylalanine was significantly accumulated, which indicated that the phenylpropane metabolic pathway in SP2 plants was affected. Current studies have shown that the phenylpropane biosynthetic pathway was activated under various abiotic stress conditions (drought, heavy metals, salinity, high/low temperature and ultraviolet radiation), thereby inducing the accumulation of various flavonoids (Sharma et al., 2019). In this study, some metabolites and proteins involved in the metabolism of phenylpropane during the TLS and TS stages of SP2 plants has changed significantly (Table 8 and Figures 9E,F). This confirmed that the impact of space flight on rice seeds lasted until the F2 generation. In addition, a large number of flavonoids were accumulated in this study, including APIIN, Oenin, Rutin, Kuromanin, Orobol, Formononetin, Malvidin 3-*O*-glucoside cation and Peonidin 3-galactoside cation. The accumulation of flavonoids in the cytoplasm can effectively decompose ROS generated by abiotic stress, and after the oxidation of flavonoids ends, ascorbic acid-mediated flavonoids were reconverted into primary metabolites and enter cell metabolism (Hernández et al., 2009). Our research results suggested that after rice seeds fly through space, there was still an oxidative stress effect in the F2 generation plants, and the F2 generation plants may also synthesize flavonoids to achieve the balance of ROS in the body.

**TABLE 8:** Differentially expressed proteins and metabolites related to phenylpropanoid biosynthesis pathway and flavonoid synthesis metabolism.

Uniprot ID	Description	Fold change		Adjusted *P*-value	
		TLS	TS	TLS	TS
Q10CU9	Glycosyl hydrolase family 3	1.41		1.2836E-03	
A0A0P0YAL3	Phenylalanine ammonia-lyase	1.3	1.35	4.0609E-02	4.5000E-02
Q84YK7	Beta-glucosidase 27	1.3		3.0213E-02	
A2ZEX7	Chalcone synthase 1	0.77	1.6	1.8876E-02	6.4225E-04
A2WS12	Aldehyde dehydrogenase		1.24		1.0564E-02
A2XPI4	CYP 450 93A2		1.44		2.5489E-03
Q7G602	Flavonoid 3′-hydroxylase		1.31		2.6334E-03
A2XNF0	Chalcone-flavonone isomerase		1.3		6.3815E-03
A3C6F1	CYP 450 A81		0.68		6.9292E-03
B8AXU3	Beta-glucosidase 19	0.73	0.67	2.6617E-02	1.0564E-02
**Metabolites**					
	4-Hydroxycinnamic acid	2.4	0.43	3.1061E-09	1.1828E-03
	Ferulic acid	2.16	1.59	2.3178E-08	3.2950E-04
	APIIN	1.57	5.2	6.1736E-07	1.1729E-04
	Malvidin 3-*O*-glucoside cation	1.22	1.5	7.4074E-04	2.0241E-02
	Peonidin 3-galactoside cation	1.2	1.5	2.1901E-04	2.4146E-02
	Chlorogenic acid		4.4		6.6984E-06
	p-Coumaryl alcohol		2.91		1.1109E-04
	Oenin		2.61		1.1157E-02
	Kaempferol	0.25	2.34	7.1541E-09	3.6814E-02
	Hesperetin 7-*O*-neohesperidoside	0.19		1.2644E-09	
	Rutin		2.01		4.6557E-02
	Orobol		1.84		1.5832E-03
	Sinapate		1.62		4.1766E-02
	Kuromanin		1.34		1.1766E-02
	Formononetin		1.33		1.4771E-03

*Blue represents down-regulated.*

*Yellow represents up-regulated.*

## Conclusion

**FIGURE 11:**
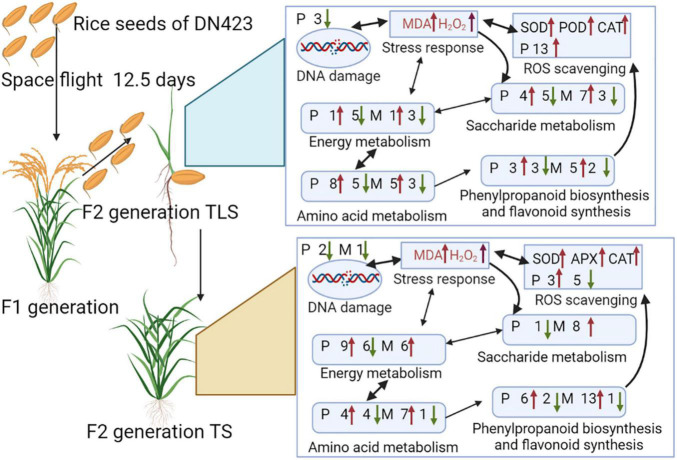
Mechanisms of memory in response to space flight stress in F2 generation rice. *P* represent proteins; M represent metabolites; numbers indicate the amount of proteins and metabolites; red and green arrows represent increase and decrease, respectively.

Overall, the F2 generation plants of rice still retained the stress of space flight to seeds, and this memory was caused by the instability of the rice genome after spaceflight. The memory of spaceflight stress induced the accumulation of ROS in the F2 generation plants, and the F2 generation plants-maintained ROS homeostasis by rebuilding their own metabolic pathways to adapt to the effects of spaceflight. As a signal molecule, ROS interacted with sugar signal pathways to mediate changes in amino acid metabolism, energy metabolism, and phenylpropane metabolism (Figure 11). These pathways helped maintain the homeostasis of ROS in plants. Moreover, the metabolic process of phenylpropane induced the biosynthesis of flavonoids, which were important for regulating ROS homeostasis (Figure 11). This study was the first one to combine metabolomics and proteomics methods to confirm that the effects of space flight on rice seeds lasted until the F2 generation, and the ability of F2 generation plants to adapt to space flight stress by rebuilding their own metabolic network. This research provides a new perspective for the study of spatial biological effects.

## Data Availability Statement

The original contributions presented in this study are included in the article/Supplementary Material, further inquiries can be directed to the corresponding author.

## Author Contributions

WL and JC designed the study. DZ conducted the experiments and analyzed the data. DZ and YY wrote the manuscript. HZ, CS, and CD revised the manuscript. SG conducted the field planting of experimental materials. DC provided the experimental site. YS guided the experiment. All authors read and approved the manuscript in its final form.

## Conflict of Interest

The authors declare that the research was conducted in the absence of any commercial or financial relationships that could be construed as a potential conflict of interest.

## Publisher’s Note

All claims expressed in this article are solely those of the authors and do not necessarily represent those of their affiliated organizations, or those of the publisher, the editors and the reviewers. Any product that may be evaluated in this article, or claim that may be made by its manufacturer, is not guaranteed or endorsed by the publisher.

## Footnotes

1
https://www.uniprot.org/

2
http://www.metaboanalyst.ca/

3
http://www.kegg.jp/

## Abbreviations

APX: Ascorbate peroxidase
CAT: Catalase
CK: F2 generation control group
DAPs: Differentially Abundant Proteins
DEMs: Differential metabolites
DN423: Dongnong423
EL: Electrolyte leakage rate
CK: F2 generation control group
EL: Electrolyte leakage rate
iTRAQ: Isobaric tags for relative and absolute quantification
PCA: Principal component analysis
POD: Peroxidase
qRT-PCR: Real-time quantitative PCR
ROS: Reactive oxygen species
SOD: Superoxide dismutase
SJ-10: ShiJian-10 retractable satellite
SSC: Soluble sugar content
TCA: Tricarboxylic acid cycle
TLS: Three-Leaf Stage
TS: Tillering Stage.
