# Supplementary material for: Combining Proteomics and Metabolomics to Analyze the Effects of Spaceflight on Rice Progeny
Source: Front Plant Sci. 2022 Jun 21;13:900143. doi: 10.3389/fpls.2022.900143 (PMC9253829; doi:10.3389/fpls.2022.900143)
Supplement: Supplementary file 1 [file Data_Sheet_1.docx]

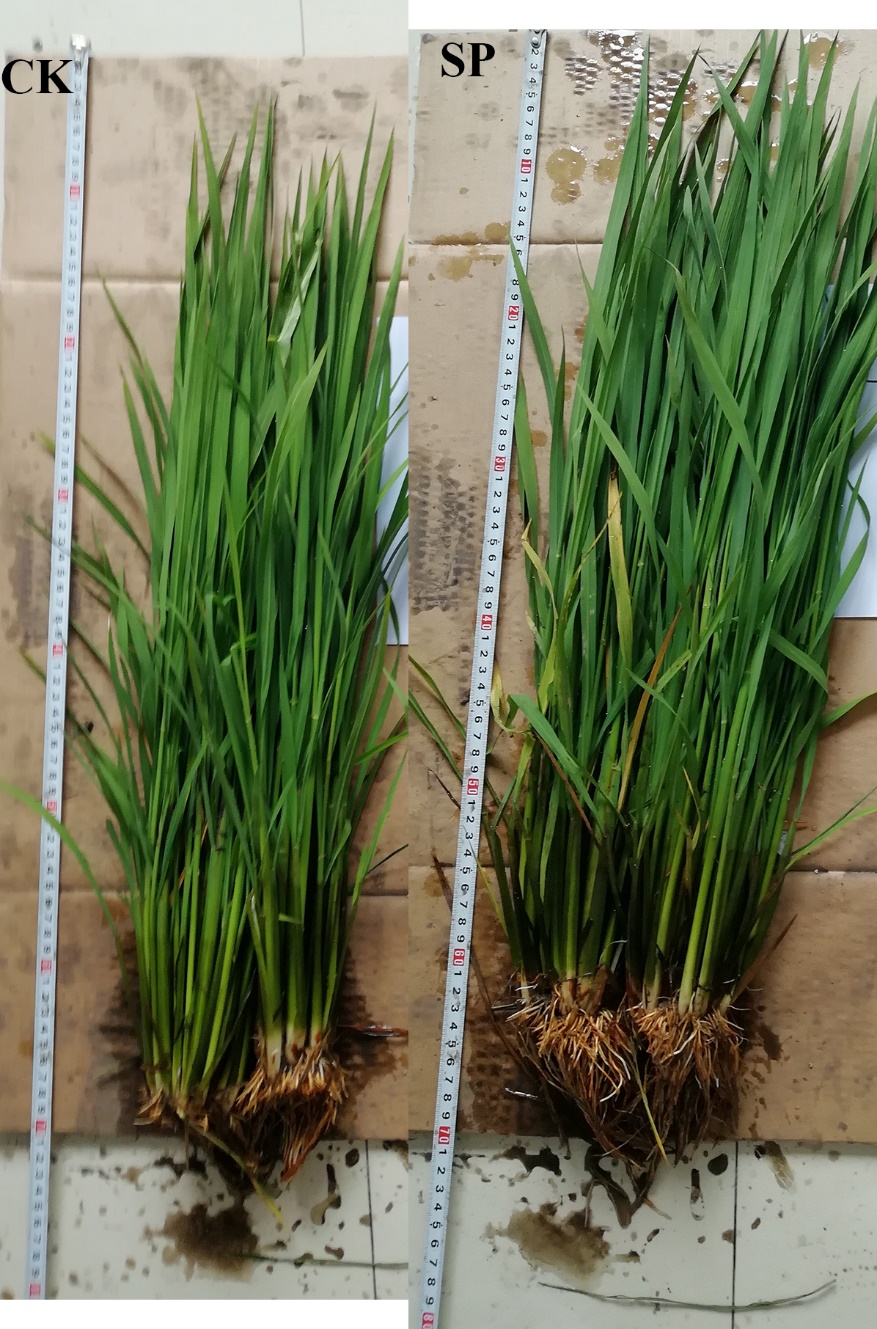


Fig. S1. Photographs of progeny rice plants in TS.


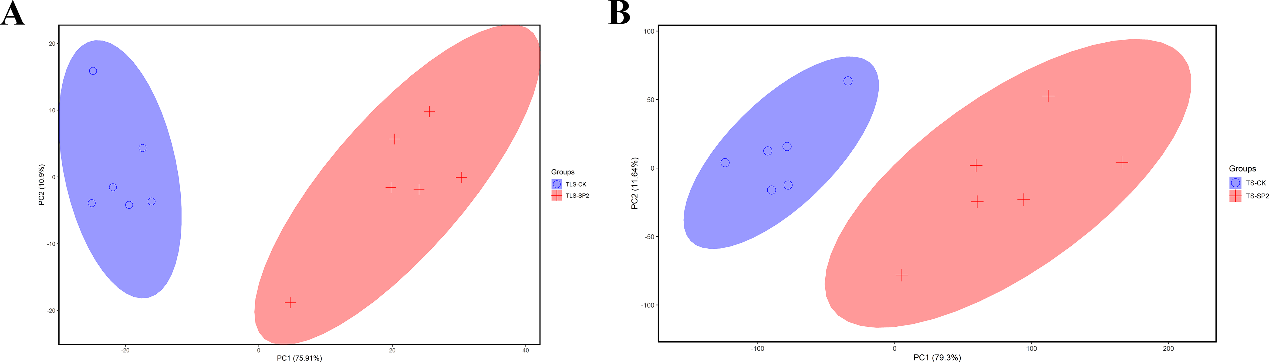


Fig. S2. PCA score map of different rice sample groups based on UHPLC-QTOF/MS data. (A) Rice samples at the TLS, (B) Rice samples at the TS; TLS and TS represent three leaf period and tillering stage.


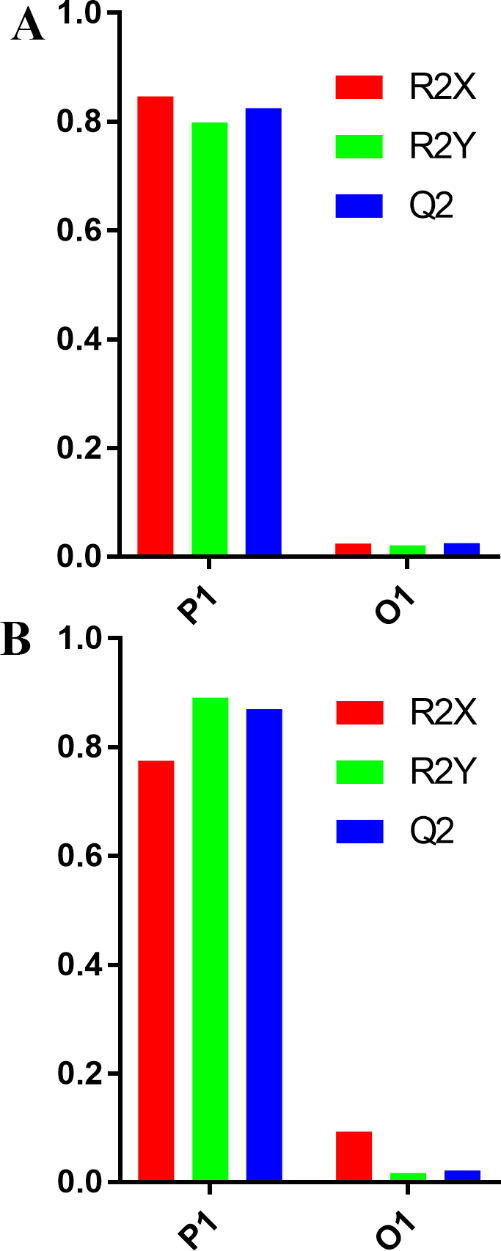


Fig.S3 Overview of orthogonal PLS-DA Analysis Models for Metabolites

CK represent control group. SP2 represent the offspring of rice after spaceflight.


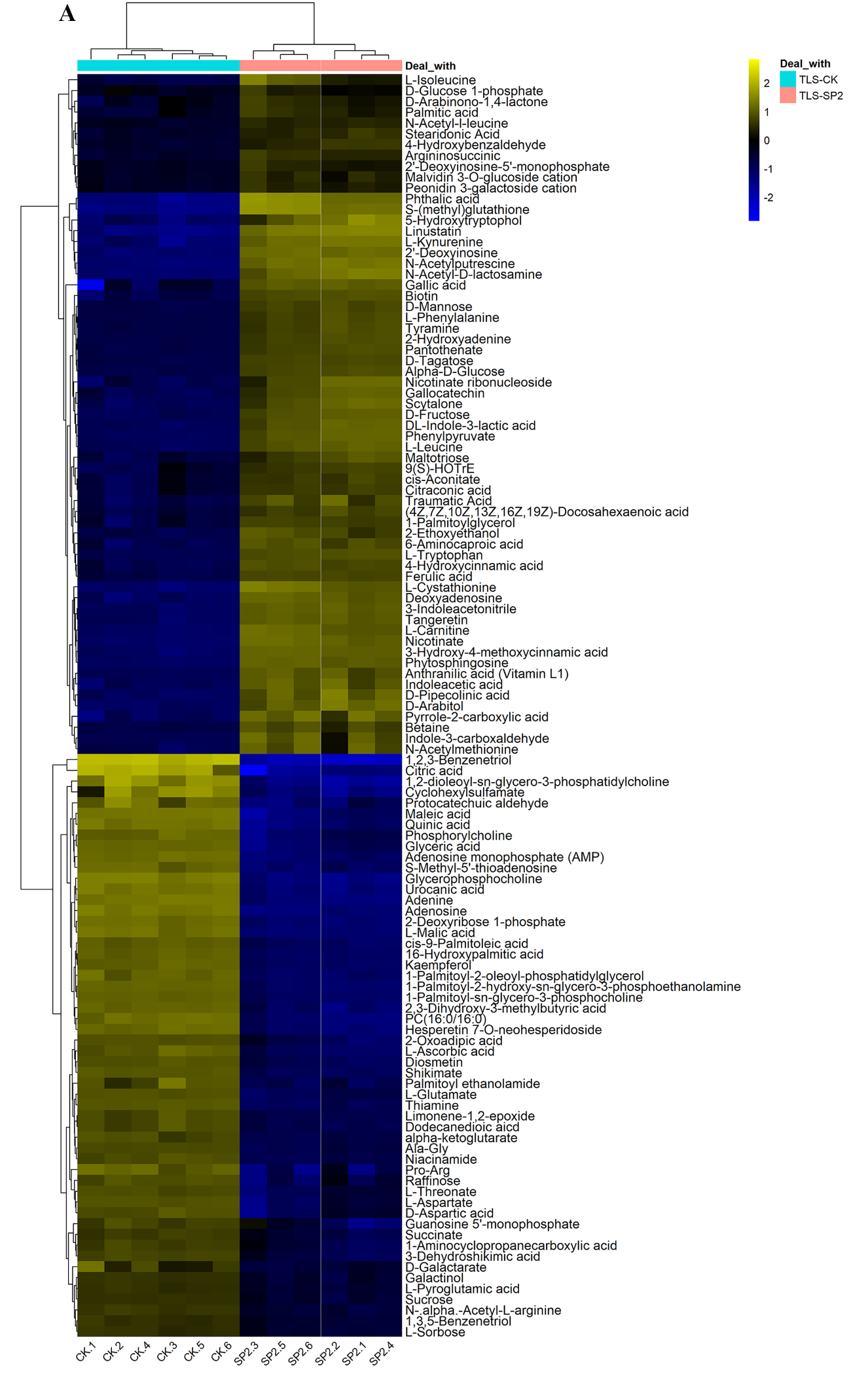


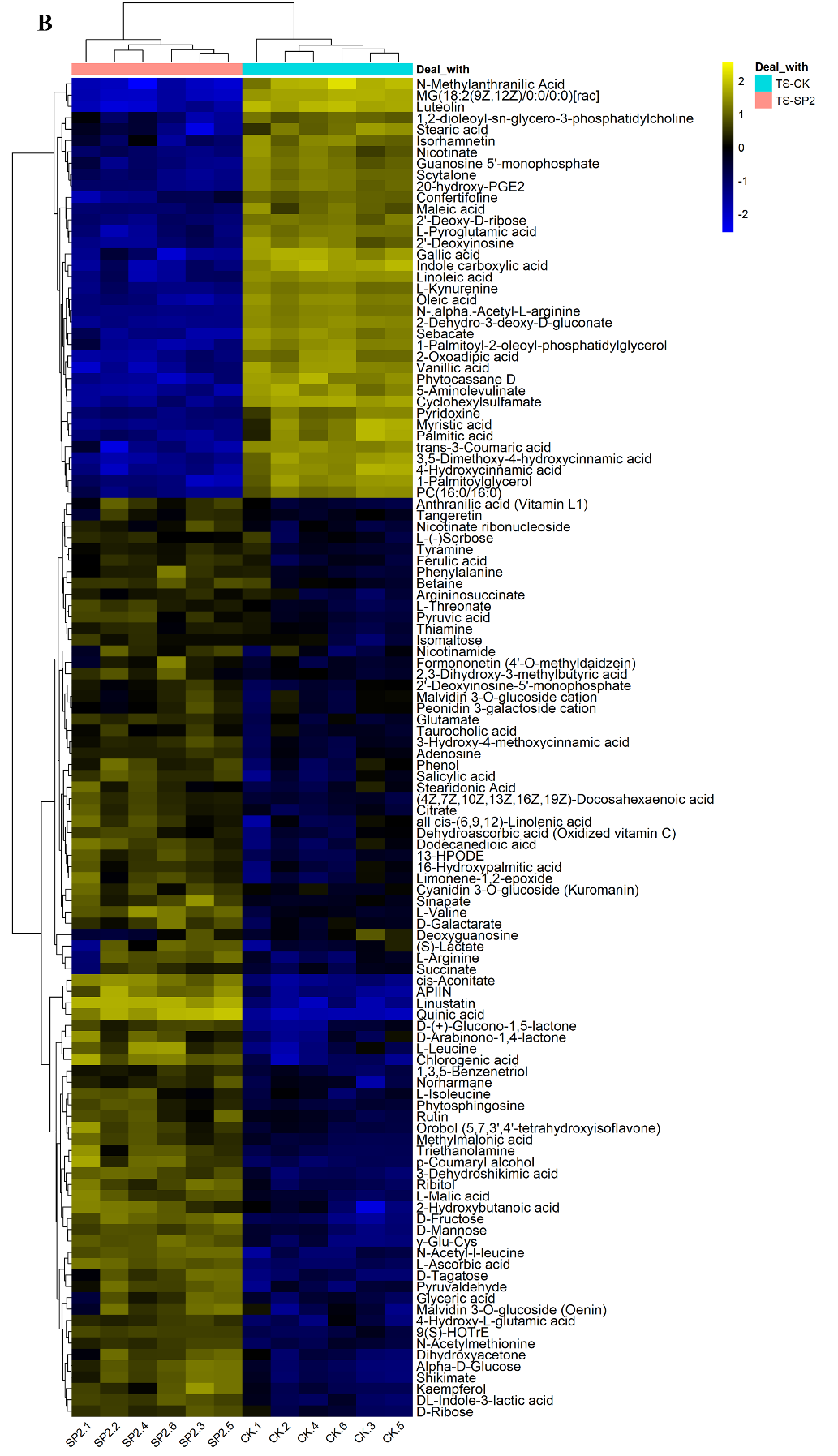


Fig. S4. Results of hierarchical cluster analysis of changed metabolites pools. Hierarchical trees were drawn based on the detected changed metabolites in leaves of rice, (A) TLS, (B) TS. Columns correspond to the repetition between different treatments, while rows represent different metabolites. yellow and blue colors indicate increased and decreased metabolite concentrations, respectively.
