# Supplementary material for: Combining Proteomics and Metabolomics to Analyze the Effects of Spaceflight on Rice Progeny
Source: Front Plant Sci. 2022 Jun 21;13:900143. doi: 10.3389/fpls.2022.900143 (PMC9253829; doi:10.3389/fpls.2022.900143)
Supplement: Supplementary file 2 [file Table_1.docx]

**Table S1. Primers used in the qRT-PCR analysis.**

| UniProt ID | name | Forward primer (5’–3’) | Reverse primer (5’–3’) |
| --- | --- | --- | --- |
| A2XTH3 | Peroxidase | ACGGCGAGATCAGGATGAAC | ATCACATCACATCGATCGGC |
| B8AXU3 | beta-glucosidase 19 | GGACTGCGGCTTTCCAGTAT | TCCCGGATTCCTCCATGAGT |
| A6N0U5 | Ndufa8 | GAACGTGGCCTTCCTCAACT | CTTGGGGCACTTCTGGTGAA |
| Q6ZJ19 | Ndufs5 | TTCAGCGAGTGTATGAGCCG | GGAACTCCTTGGAGTGTGGG |
| A2WXB2 | Fructose-1,6-bisphosphatase | AGAGTGGTGCGGAGATGGA | TACGGTGGACATCAGCAACC |
| Q7XKW5 | L-threonine aldolase 1 | GTTCCATCGGGGTTGTTCCT | TGGTCGGATAATACAGCGCC |
| A0A0P0YAL3 | Phenylalanine ammonia-lyase | ACATCAACCCACTGCTGGAG | CGTCAGACGTACACCTCGTC |
| A2ZEX7 | Chalcone synthase 1 | GCCGGTGACCTGGTGAATTA | ACATGTTGGGGTTCTCCTGC |
